# The gill-associated microbiome is the main source of wood plant polysaccharide hydrolases and secondary metabolite gene clusters in the mangrove shipworm *Neoteredo reynei*

**DOI:** 10.1371/journal.pone.0200437

**Published:** 2018-11-14

**Authors:** Thais L. Brito, Amanda B. Campos, F. A. Bastiaan von Meijenfeldt, Julio P. Daniel, Gabriella B. Ribeiro, Genivaldo G. Z. Silva, Diego V. Wilke, Daniela T. de Moraes, Bas E. Dutilh, Pedro M. Meirelles, Amaro E. Trindade-Silva

**Affiliations:** 1 Drug Research and Development Center, Department of Physiology and Pharmacology, Federal University of Ceara, Fortaleza, Ceara, Brazil; 2 Institute of Biology, Federal University of Bahia, Salvador, Bahia, Brazil; 3 Theoretical Biology and Bioinformatics, Utrecht University, Utrecht, Netherlands; 4 Computational Science Research Center, San Diego State University, San Diego, California, United States of America; 5 National Institute of Metrology, Quality and Technology, Rio de Janeiro, Brazil; 6 Centre for Molecular and Biomolecular Informatics, Radboud University Medical Centre, Nijmegen, The Netherlands; 7 National Institute of Science and Technology in Interdisciplinary and Transdisciplinary Studies in Ecology and Evolution (INCT IN-TREE), Federal University of Bahia, Salvador, Brazil; USDA Forest Service, UNITED STATES

## Abstract

Teredinidae are a family of highly adapted wood-feeding and wood-boring bivalves, commonly known as shipworms, whose evolution is linked to the acquisition of cellulolytic gammaproteobacterial symbionts harbored in bacteriocytes within the gills. In the present work we applied metagenomics to characterize microbiomes of the gills and digestive tract of *Neoteredo reynei*, a mangrove-adapted shipworm species found over a large range of the Brazilian coast. Comparative metagenomics grouped the gill symbiont community of different *N*. *reynei* specimens, indicating closely related bacterial types are shared. Similarly, the intestine and digestive gland communities were related, yet were more diverse than and showed no overlap with the gill community. Annotation of assembled metagenomic contigs revealed that the gill symbiotic community of *N*. *reynei* encodes a plethora of plant cell wall polysaccharides degrading glycoside hydrolase encoding genes, and Biosynthetic Gene Clusters (BGCs). In contrast, the digestive tract microbiomes seem to play little role in wood digestion and secondary metabolites biosynthesis. Metagenome binning recovered the nearly complete genome sequences of two symbiotic *Teredinibacter* strains from the gills, a representative of *Teredinibacter turnerae* “clade I” strain, and a yet to be cultivated *Teredinibacter* sp. type. These *Teredinibacter* genomes, as well as un-binned gill-derived gammaproteobacteria contigs, also include an endo-β-1,4-xylanase/acetylxylan esterase multi-catalytic carbohydrate-active enzyme, and a trans-acyltransferase polyketide synthase (trans-AT PKS) gene cluster with the gene cassette for generating β-branching on complex polyketides. Finally, we use multivariate analyses to show that the secondary metabolome from the genomes of *Teredinibacter* representatives, including genomes binned from *N*. *reynei* gills’ metagenomes presented herein, stands out within the Cellvibrionaceae family by size, and enrichments for polyketide, nonribosomal peptide and hybrid BGCs. Results presented here add to the growing characterization of shipworm symbiotic microbiomes and indicate that the *N*. *reynei* gill gammaproteobacterial community is a prolific source of biotechnologically relevant enzymes for wood-digestion and bioactive compounds production.

## Introduction

The Teredinidae, a family of obligate xylotrepetic (wood-boring) and xylotrophic (wood-feeding) mollusks, evolved from an ancestor lineage within the eulamellibranch order Myoida, and succeeded in exploiting wood as new food source, most probably by concomitantly acquiring cellulolytic bacterial symbionts [1]. These animals underwent dramatic body shape adaptations to thrive as wood-feeders including greatly reduced and burrowing-specialized valves (shells), and a wood-sheltered worm-like body plan protruding from their shells, which led to their common name of shipworms [2].

Shipworm bacterial symbiotic communities are formed by cultivated and as yet uncultivated closely related gammaproteobacteria, located intracellularly in bacteriocytes within the gills (ctenidia) tissue [3–6]. The first cultivated shipworm symbiont is the cellulolytic and nitrogen-fixing bacterium *Teredinibacter turnerae*, isolated from a great variety of Teredinidae species from all over the globe [7,8]. The complete genome of one strain of *T*. *turnerae*, T7901, revealed an arsenal of enzymes specialized in breaking down woody material, reinforcing the hypothesis that shipworm gammaproteobacterial symbionts play a critical role in supporting host nutrition [9]. Indeed, two seminal works provided strong evidence for this hypothesis: firstly multi isotopic mass spectroscopy (MIMS) combined with transmission electron microscopy (TEM) showed, in *Lyrodus pedicellatus*, that *T*. *turnerae* cells could fix atmospheric N_2_ *in symbio*, and then transfer nitrogenized compounds to their host [10]. Secondly, a combination of metagenomics and proteomics of *Bankia setacea*, showed that wood-specialized hydrolytic enzymes secreted by the symbiotic community are, selectively transported from the symbionts on the gill, to the host’s cecum, the primary site of wood digestion [11].

Characterization of the *T*. *turnerae* T7901 genome also revealed that this endosymbiotic bacterium contains a diversity of Biosynthetic Gene Clusters (BGCs) for secondary metabolites comparable to those observed in free-living *Streptomyces* species known for their proficiency at secondary metabolite production [9]. Indeed, compounds of the tartrolon family of boronated antibiotic [12], and a novel triscatecholate siderophore called turnerbactin [13], were purified from chemical extracts of *T*. *turnerae* cultures and the respective biosynthetic routes identified and characterized by retro-biosynthetic and gene mutation approaches. Moreover, both BGCs were proven to be expressed *in symbio* and signatures of these compounds were detected by mass spectroscopy of shipworms whole animal extracts [12,13]. Such results fueled a hypothesis that bioactive secondary metabolites could also play a role in supporting the symbiosis by providing chemical defense to bolster holobiont fitness [14], and/or mediate competition/specialization to shape the distribution of bacterial types within the gills. Fluorescence *in situ* hybridization (FISH) and confocal microscopy surveys of tissues from several shipworm species provided evidence supporting both these hypothesis, showing that i) symbionts are retained in bacteriocytes—specialized structures within the host gill and ii) tissues of the shipworm digestive system are virtually sterile (cecum) or contain a discrete microbial community (intestine) [11,15].

Recently, the gill symbiotic community of the giant shipworm *Kuphus polythalamia*, a rare species that burrows into mud and sediment instead of wood, was shown to be composed of sulfur oxidizing chemoautotrophic gammaproteobacteria instead of Teredinibacter-related cellulolytic types [16]. These findings, along with singular morphological features, suggested this bivalve is a chemoautotrophic relative of xylotrophic Teredinidae, thus adding complexity to the biology of symbiosis in the family. *Neoteredo reynei* (Bartsch, 1920) is another singular shipworm that has been reported on mangroves of the south Atlantic, including records of a large range of the 7,367 km long Brazilian coastline, from the States of Pará, at the north of the country, to Santa Catarina, at the very south. *N*. *reynei* is the only species of the genus, and can be rapidly identified by a unique feature: the presence of large and highly vascularized dorsal lappets at the posterior end of the animal’s body, whose function is yet to be clarified [2,17]. *N*. *reynei* is a basal member of the family and has, characteristically, unsegmented pallets, a globular (type II) stomach and an intestine that makes a loose loop forward embracing the crystalline style-sac before running backwards toward the cecum [1]. The *N*. *reynei* digestive tract also includes two digestive glands, one attributed to wood-digestion, the other to plankton-feeding, and both connected to the style-sac through ducts. In addition, *N*. *reynei* possesses a large cecum, which can reach up to 44% of the animal’s body size, and an enormous, cylindrically shaped anal canal whose aperture is controlled by a strong muscular sphincter [18]. *N*. *reynei* gills extend from the base of the siphons up to the posterior end of the visceral mass as demibranchs and continues as a reduced food groove at the anterior end of the animal’s body, without presenting an anterior gill, like in most other shipworm species. Therefore, it comprises only 20% of *N*. *reynei* body length, a much shorter proportion when compared to other shipworm species. Such reduced gills combined with a large cecum and anal canal, both continuously filled with wood particles, were taken as anatomical evidences that *N*. *reynei* diet is primarily wood [2,17,18]. Previous culturing efforts lead to isolation of *T*. *turnerae* strains from *N*. *reynei* gill homogenate. One of these strains, CS30, displayed antimicrobial activity against a spectrum of gram positive and negative bacteria [19]. However, to date, the diversity and biotechnological potential of *N*. *reynei* gill symbiotic microbiome had yet to be explored by culture independent methods.

Herein, we applied metagenomics to explore the bacterial communities present at the digestive glands, intestine, and gill symbiotic-site of the *N*. *reynei*. Two symbiotic bacterial genome bins were recovered and analyzed for their capability for producing hydrolytic enzymes and bioactive secondary metabolites. Our findings reinforced the importance of the shipworm symbiotic system as a subject of study and discovery.

## Material and methods

### Specimens collection

Three whole adult specimens of *N*. *reynei* shipworm were collected from decaying wood in the Coroa Grande mangrove area at Sepetiba Bay, Rio de Janeiro State, Brazil (22.91°S, 43.87°W) on December 5, 2014, and transported to the Laboratory of Biotechnology at the National Institute of Metrology, Quality and Technology (Inmetro, RJ) in sterilized glass jars. Vouchers containing animals’ identifying characters syphon and pallets were deposited in the Malacological collection “Professor Henry Ramos Matthews”–Series B of the Federal University of Ceara, under the register number CMPHRM 5974B. Sampling was performed under the authorization of the Brazilian Environmental Agency, Instituto Chico Mendes de Conservação da Biodiversidade (SISBIO license n^o^ 48388). The genetic resources of the present study were accessed under the authorization of the Brazilian National System for the Management of Genetic Heritage and Associated Traditional Knowledge (SisGen permit n^o^ A2F0DA0).

### DNA extraction, sequencing, annotation, and statistics

Digestive glands, intestines and gill tissues were dissected from freshly collected animals, snap frozen and ground in liquid nitrogen, then processed for metagenomic DNA extraction using 2% CTAB lysis buffer, phenol-chloroform-isoamyl alcohol (25:24:1) deproteinization, and the columns from PowerSoil DNA Isolation Kit (MO BIO Laboratories, Carlsbad, CA, USA) for final purification, as previously described [20]. For each specimen, the two digestive glands were combined prior to grinding for DNA purification. Additionally, two independent DNA purifications were performed from pulverized intestine and gills tissues (replicates), totaling five metagenomic DNA sample per shipworm specimen. Metagenomic DNA samples were quantified using the NanoDrop spectrophotometer (Thermo Fisher Scientific Inc.) and Qubit fluorimeter (Thermo Fisher Scientific Inc.), while integrity was confirmed by gel electrophoresis. Metagenomic DNA libraries were prepared from the fifteen high-quality metagenomic DNA samples, using a *Nextera XT DNA Sample Preparation Kit* (Illumina), as recommended by the manufacturer. Metagenomic libraries were sequenced using the 600-cycle (300 bp paired-end runs) *MiSeq Reagent Kits v3* chemistry (Illumina) with a MiSeq Desktop Sequencer (Illumina) at the Center for Genomics and Bioinformatics (CeGenBio) of the Drug Research and Development Center (NPDM), at the Federal University of Ceara, Brazil. Raw sequence data were submitted to the MG-RAST server (version 4.03) [21] for paired-end joining, quality control, and automated annotation pipeline. Taxonomic annotations were performed using RefSeq, and functional signatures were retrieved with both Clusters of Orthologous Groups (COG) and Subsystems technology databases using the default cutoffs settings. Taxonomical and functional signatures were submitted to comparative metagenomics using R packages and the Statistical Analysis of Metagenomic Profiles (STAMP) software package, version 2.1.3[22]. Multivariate statistical analyzes were performed with R using the following unsupervised learning techniques: i) hierarchical clustering, with Ward grouping method on a Euclidean distance matrix, ii) PCA biplot, and iii) supervised/unsupervised random forest at R, as previously reported [23]. For hierarchical clustering, cluster significance was accessed by bootstrap resampling using pvclust algorithm[24]. At STAMP, “two groups” comparisons were conducted considering the two main clades obtained by unsupervised hierarchical clustering. For that, the prokaryotic taxonomic (Class level of RefSeq) and functional (Level 2 of the Subsystems technology) features of each group were retrieved from MG-RAST and compared using two-sided Welch’s t-Test with confidence interval of 0.95 and considering Benjamini-Hochberg False Discovery Rate corrected P-values (q-values) < 10^−5^ as significantly relevant.

### Metagenome assembly, cross-comparison and contig annotation

For annotation-independent cross-assembly comparison between the fifteen metagenomic samples generated in this study we used crAss tool [25]. First, all paired-end joined quality controlled metagenome reads of all tissues and specimens were assembled together using SPAdes 3.8 in—meta mode, and k-mer sizes of 21, 33, 55, and 77 [26]. Reads were mapped back to contigs using Bowtie2 [27] and the SAM file was used on crAss, which computes the abundance of reads from each metagenome contributing to form each contig in the cross assembly. The coverage of each metagenomes in contigs was used to build a distance matrix that was displayed in the form of a cladogram [25]. The tissue-specific contigs retrieved from gills, intestine and digestive glands datasets were taxonomically annotated with the contig annotation tool (CAT) [28] which predicts protein-coding genes on a contig, maps these to NCBI's non-redundant protein database (NR), and subsequently employs a last common ancestor algorithm to give a conservative estimate of taxonomy for the contig. CAT was run with the b1 parameter set to 10, and the b2 parameter set to 0.5. Prodigal 2.6.3 [29] and Diamond 0.9.14 [30] were employed for protein calling and database mapping, respectively. NR was downloaded on November 23, 2017.

### Symbiotic genome binning

Metagenomic reads were cross-assembled for each tissue with SPAdes 3.8.0 in—meta mode [26]. Tissue-specific contig sets were each binned into draft genome sequences using MetaBAT 0.26.3 [31] that exploits signatures that are typical of contigs belonging to the same genome, including the nucleotide usage and the abundance of the contigs in the different samples. In order to get contig abundances, reads from all the samples were mapped to the tissue-specific assemblies with Burrows-Wheeler Aligner (BWA) 0.7.12 with the BWA-MEM algorithm[32]. Mappings were converted to the binary BAM format with SAMtools 0.1.19 [33] and depth files were created with the script jgi_bam_summarize_contig_depths that is supplied with MetaBAT. Binning with MetaBAT was performed with the sensitive setting, after tests with other presets showed this to give the best results in terms of bin completeness and contamination. Bin completeness, contamination, and strain heterogeneity were assessed using CheckM 1.0.7 [34] based on the presence and absence of sets of single-copy marker genes. CheckM was run in the lineage specific work flow, which places the bins in a reference tree and based on that placement searches for lineage-specific marker genes. Completeness, contamination, and strain heterogeneity were assessed on the lowest level of placement (node) in the CheckM backbone tree.

### Symbiotic genome functional annotation and multivariate analysis

Nearly complete genomes gills.bin.1 and gills.bin.4, as well as other related gammaproteobacterial genomes, including representatives of the Cellvibrionaceae family, and more specifically, the *Teredinibacter* clade, were automatically annotated at the RAST server [35] under default settings. Carbohydrate-active enzymes (CAZymes) were annotated using the dbCAN web server [36]. Results were filtered for E-value of 1e^-5^ and domain coverage fraction > 0.7. Using such parameters, reannotation of *T*. *turnerae* T7901 genome leads to recover of 99% of the CAZy domains, with false positive and false negative rates of ~8 and ~7% respectively. Natural products Biosynthetic Gene Clusters (BGCs) were annotated using antiSMASH bacterial version [37] with RAST GenBank generated files as input and default settings. The genes composing the resistome (i.e. genes related to antibiotic resistance) were annotated and quantified with RGI (Resistome Genes Identifier) tool of the Comprehensive Antibiotic Resistance Database (CARD) [38], under the *discovery* criteria for detection of perfect, strict and loose hits. We then performed Principal Component Analysis (PCA) to cluster the selected genomes according their diversities of BGCs and resistance genes, and supervised Random Forest (RF) [39] to highlight the fifteen most influential vectors in each case, using ggbiplot R library (Vu, 2011) to plot the results. The number of variables in PCA was reduced by supervised RF to, with higher mean accuracy using the R library [40]. To test if the relative abundance of resistome and metabolome related genes were significantly different between free-living and host-associated bacteria we performed Kruskal-Wallis non-parametric test using the kruskal.test() function in R and considered p-value lower than 0.05 significant. We tested each category independently and adjusted the p-value using the Bonferroni adjust method with the p.adjust()function in R.

### Phylogenetic analysis

The two high quality genome bins were placed in a phylogeny with closely related Gammaproteobacteria based on the 43 phylogenetically informative marker genes that CheckM uses to assess bin placement in its own backbone tree (S6 Table in [34]). All genomes from the family Cellvibrionaceae were downloaded from the PATRIC (Pathosystems Resource Integration Center, [41] genome database, to our knowledge the largest publicly available prokaryotic genome database. One genome per family of Halieaceae, Porticoccaceae, and Spongiibacteraceae was added to together serve as outgroups. Genomes were downloaded based on the genome_lineage file on the PATRIC server that was downloaded on December 20, 2017. One Cellvibrionaceae genome present in the genome_lineage file, *Cellvibrio mixtus* strain PSBB022, could not be retrieved from the PATRIC server. CheckM was run on all the genomes to identify the 43 marker genes, translated sequences were individually realigned after dealignment with Clustal Omega 1.2.3 [42] and subsequently concatenated, filling gaps if a gene was not found in a genome. A maximum-likelihood tree was inferred with RAxML 8.2.9 [43] using the PROTCATLG model and 100 rapid bootstraps (random seeds -p and -x were both set to 12345), and the best scoring tree was visualized with iToL [44]. Additionally, the partial nucleotide sequences of the protein-coding gene *rpoB* (1,020 bp) and *gryB* (1,053 bp) were retrieved from a previously determined set of 25 *T*. *turnerae* isolates [8], and aligned with respective gene fragments from binned genomes gills.bin.1 and gills.bin.4 with Molecular Evolutionary Genetics Analysis (MEGA) software version 6.0[45], using the built-in ClustalW program with default settings for “*codons*” (protein-coding sequences). Maximum likelihood fits for 24 different nucleotide models were evaluated for each gene and best-fit model of evolution selected according to the lowest Bayesian Information Criteria (BIC) scores. Neighbor-joining phylogenies were reconstructed employing Kimura 2-parameter nucleotide substitution model improved with 5 discrete gamma categories for a total of 1000 bootstraps.

## Results

### Microbial communities in the gill and gut of *Neoteredo reynei*

To extend knowledge regarding the role of shipworm symbiotic bacteria in wood digestion and host defense, we performed functional metagenomics of *N*. *reynei* digestive gland, intestine, and gill tissues (see methods). Shotgun sequencing of fifteen metagenomes samples (S1 Table), generated 21,548,783 reads, from which approximately 92% (3.94 Gb) passed the quality control and annotation pipeline of the MG-RAST server. Approximately 1.2, 55.7 and 3.8% of the quality proofed data matched hits for ribosomal RNA, protein, and protein with known functional coding genes respectively (S1 Table).

**Fig 1:**
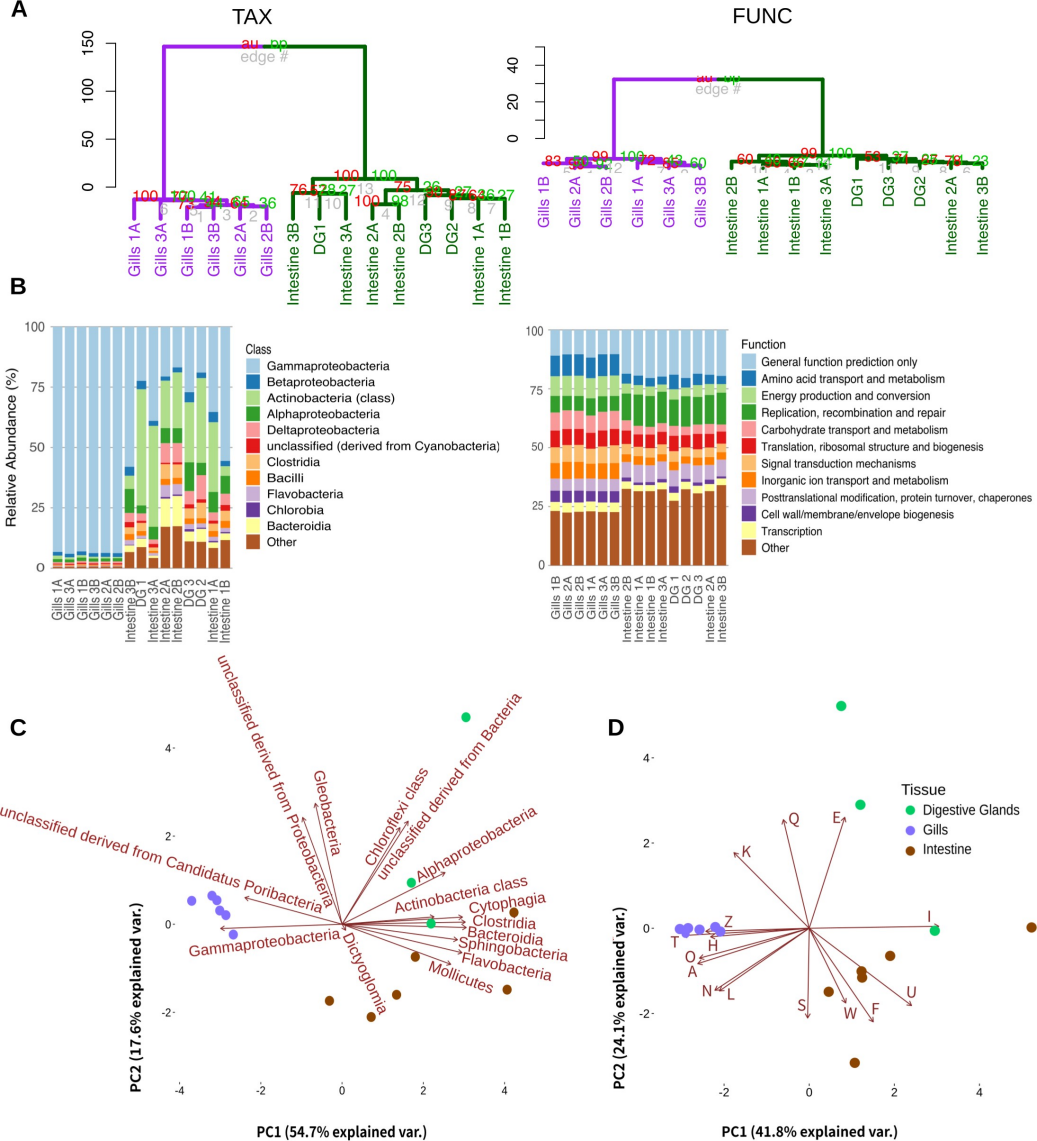
Multivariate analysis of metagenomes microbial taxonomical and functional features. A) Unsupervised hierarchical clustering of taxonomical (TAX) and functional (FUNC) features. Clades formed by gill samples and intestine plus digestive gland samples are highlighted respectively in purple and dark green. The pvclust AU (Approximately Unbiased) and BP (Bootstrap Probability) p-values are shown at clades nodes in red and light green respectively. B) Relative abundances of metagenome bacterial classes and functions respectively signed with RefSeq and COG (Level 2) databases. C) Multiple dimensional scale (MDS) plot of *N*. *reynei* metagenomics samples using distances and the fifteen most important bacteria classes (RefSeq database) and functions (COG database at Level 2) vectors as calculated from unsupervised random forest. D) Digestive gland, intestine and gill samples are shown as green, brown and purple dots respectively. Clusters of Orthologous Groups are classified into functional categories as follow: A—RNA processing and modification; E—Amino acid transport and metabolism; F—Nucleotide transport and metabolism; H—Coenzyme transport and metabolism; I—Lipid transport and metabolism; K–Transcription; L—Replication, recombination and repair; N—Cell motility; O—Post-translational modification, protein turnover, and chaperones; Q—Secondary metabolites biosynthesis, transport, and catabolism; S—Function Unknown; T—Signal transduction mechanisms; U—Intracellular trafficking, secretion, and vesicular transport; W—Extracellular structures; Z–Cytoskeleton.

Unsupervised multivariate analysis showed that the *N*. *reynei* gill microbial community has unique taxonomic and functional profiles (Fig 1). Hierarchical clustering of the microbial annotations formed consistent cladogram topologies, with gills samples grouping apart from digestive gland and intestine samples in two main clades (Fig 1A and 1B). Indeed, gill samples exhibited a dense but low diversity microbiome, with bacteria dominating the hits against the RefSeq database (> 98%), and the vast majority of these hits being tracked to Gammaproteobacteria (> 92%), and ultimately, to the genus *Teredinibacter* (Fig 1A, S1 Fig). Accordingly, 16S rRNA annotation against the RDP database confirmed the dominance of signatures of *Teredinibacter* in the gill microbiomes (~ 88%) (S1B Fig). In contrast to the gill, intestine and digestive gland samples presented more diverse microbiomes representing 23.8 and 28.7% of the taxonomical annotations (Fig 1A, S1 Fig).

Non-parametric multidimensional scaling (nMDS) combined with random forest biplot (Fig 1C) showed that enrichments to Gammaproteobacteria is a major vector driving the observed grouping of gill samples, while gram-positive (Actinobacteria, Flavobacteriia, Clostridia), gram-negative (Alphaproteobacteria, Cloroflexia, Bacteroidia, Sphingobacteriia), and unclassified Cyanobacteria influence the grouping of digestive glands and/or intestine samples. Additionally, functions under the COG subcategories *Cytoskeleton; Cell motility*; *Coenzyme transport and metabolism*; *RNA processing and modification*; *Signal transduction mechanisms*; *Posttranslational modification*, *protein turnover*, *chaperones*; *Transcription*; and *Secondary metabolites biosynthesis*, *transport and catabolism* influenced gill samples grouping, while digestive glands and intestine microbiomes were driven by *Lipid; Amino acid*, *and Nucleotide transport and metabolism* besides *Extracellular structures*, and *Intracellular trafficking secretion and vesicular transport* (Fig 1D). The two group comparisons performed with reference to the two main clades obtained by the hierarchical clustering analysis and using taxonomical and functional microbial sequence annotations corroborate the nMDS analysis, with biologically significant enrichments detected in either gills or digestive tract (digestive glands and intestine) microbiomes closely matching the vectors driving samples grouping on nMDS plot (S2 Fig).

Although highly informative, comparing taxonomic and functional annotations confine the sequence data to the reference databases, therefore biasing the results according to the database representativeness. Considering that, we performed further annotation-independent comparative metagenomics with crAss [25], a tool that accesses (and score) similarities between metagenomes according to their shared entities (cross-contigs) after cross-assembling all sample reads. When quantitative distance formulas "reads" and "wootters" were used, in which the contig number of reads is considered as reflecting its abundance in the environment, samples grouped significantly (p<0.001) by the tissue, driven by gill samples, which clustered together regardless of the animal source (S3 Fig).

Further, we applied the Contigs Annotation Tool (CAT) algorithm to get taxonomical classification of contigs assembled for each tissue-type dataset (see Methods). Such analysis showed that gill samples contain ten times more bacterial-derived contigs (~25% of the signatures) than intestine and digestive glands (~2–3% of the signatures) (S4 Fig). Mollusca was the most prevailing taxon representing 30–38% of tissue signatures (S4 Fig). Corroborating raw reads annotations, Gammaproteobacteria dominated the gill microbial community (~87% of bacterial contigs), with *Teredinibacter* as the most abundant classified bacterial genus (~81% of gammaproteobacterial contigs). Additionally, the discrete microbiomes of digestive glands and intestine were reaffirmed as more diverse with Proteobacteria (~0.6–0.7%), Actinobacteria (~0.4–0.5%), Bacteroidetes (~0.3–0.6%) and Firmicutes (~0.3%) contigs leading the obtained microbial hits (S4 Fig).

### Gill symbiont composition

Genome binning was then performed in an effort to recover microbial genomes representing each investigated tissue microbiome. Two high quality (> 80% completeness, < 5% contamination) gammaproteobacterial genomes bins labeled as “gills.bin.1” and “gills bin.4” were generated from the gill assembly and automatically assigned as *T*. *turnerae* and *Teredinibacter* sp. by CheckM (Table 1). On the other hand, no bins presenting the required quality for further analysis (> 50% completeness, < 5% contamination) could be recovered from the digestive gland or intestine assemblies.

**Table 1:** *N*. *reynei* gill symbiotic genome bins metrics and features.

Genome bin/Features	Gills.bin.1	Gills.bin.4
**Marker lineage^a^**	s_turnerae (UID4667)	g_Teredinibacter (UID4659)
**Completeness^a^**	87.12	96.45
**Contamination^a^**	2.8	4.88
**Strain heterogeneity^a^**	0	55
**Genome size (bp)**	4418468	5127066
**# contigs**	355	207
**N50**	16757	39993
**Mean contig length (bp)**	12446	24768
**Longest contig (bp)**	97957	134446
**GC content**	51.59	53.41

Genome bins quality as accessed by the CheckM tool.

#—Number. Abbreviation: bp–base pairs.

^a^–Genome bins quality as accessed by CheckM [34]. The genome bins “marker lineage” represents the lower level tree placement (node) in the CheckM backbone tree for marker gene set inference. "Completeness" and "contamination" estimates are based on presences and absences of inferred marker gene sets. "Strain heterogeneity" represents multi-copy marker genes with close amino acid identity and is thus an indication for closely-related strains that are binned together.

**Fig 2:**
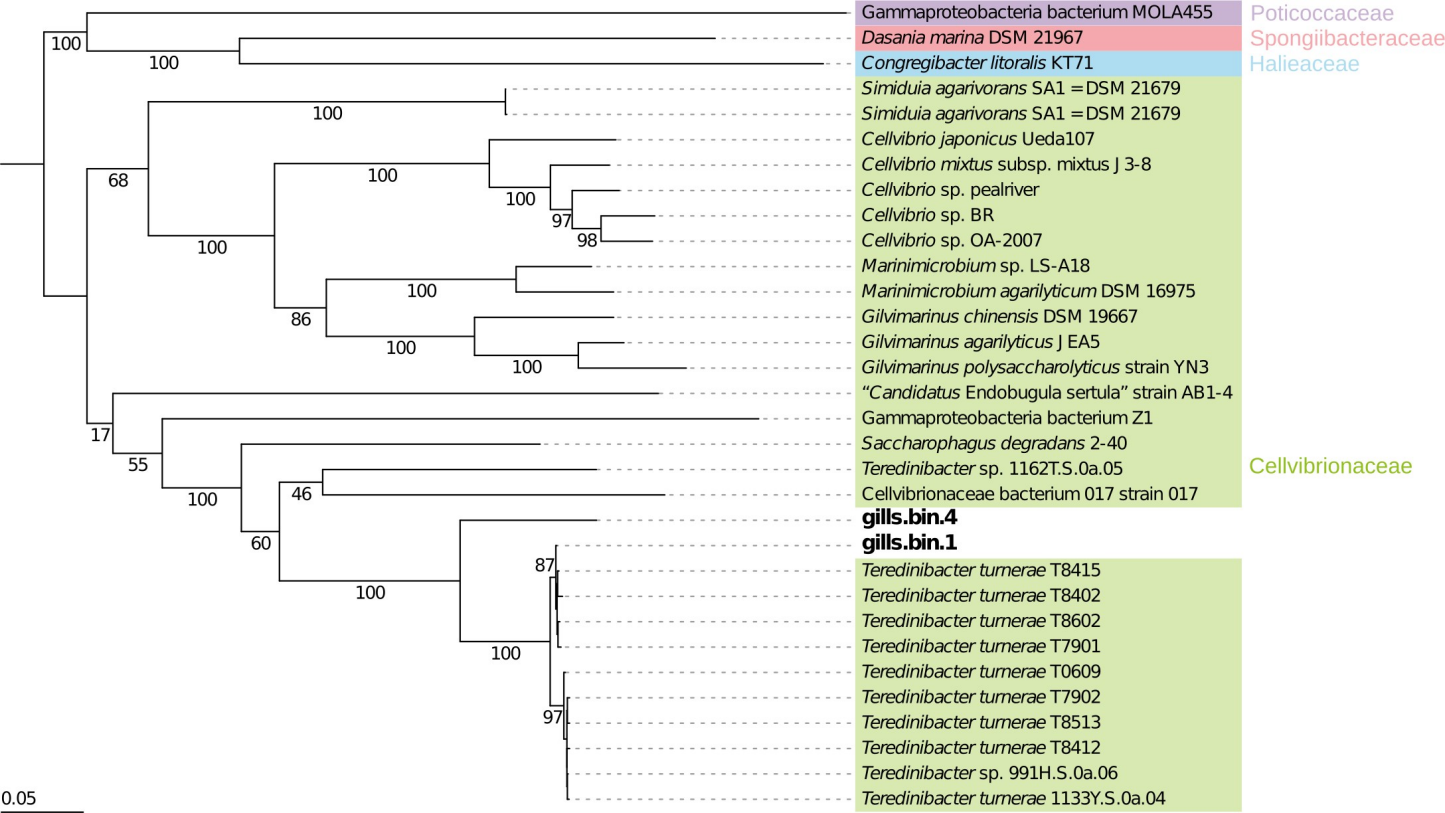
Phylogeny of two high quality Teredinibacter bins based on a concatenated alignment of 43 proteins. RAxML maximum likelihood tree based on the CAT model of rate heterogeneity and LG amino acid substitution matrix, with 100 rapid bootstraps. To improve readability, bootstraps for the *Teredinibacter turnerae* sub-clades I and II (with bootstrap values of 87 and 97 respectively) are omitted. The tree is rooted in between the Cellvibrionaceae branch and representatives of Halieaceae, Porticoccaceae, and Spongiibacteraceae. Color-coding of the families is similar to the one used by Spring and co-authors (2015). The scale bar represents 0.05 nucleotide substitutions per site.

Firstly, the taxonomic affiliations of gills.bin.1 and gills.bin.4 genome bins were checked by genome-scaled supermatrix (Fig 2) and one-protein-coding taxonomic marker phylogenies (S5 Fig). Corroborating with the CheckM taxonomical annotation, which is based on lineage-specific marker genes, all phylogenies provided highly similar tree topologies where both genome bins fell in a subclade of Cellvibrionaceae family, with gills.bin.1 grouping within the previously defined “clade 1” of *T*. *turnerae* [8] and gills.bin.4 appearing as outgroup of this species main clade (Fig 2 and S5 Fig). The detection of a *N*. *reynei* symbiotic “clade I” *T*. *turnerae* strain is in agreement with previous affiliation of a *N*. *reynei T*. *turnerae* isolate, T8508, also falling within this clade [8]. Moreover, as observed for other Teredinidae species, the present results showed that *N*. *reynei* gill symbiotic microbiota contains species in the *Teredinibacter* clade including, but not limited to, *T*. *turnerae*.

### Wood-digestion specialization in the symbiome

**Fig 3:**
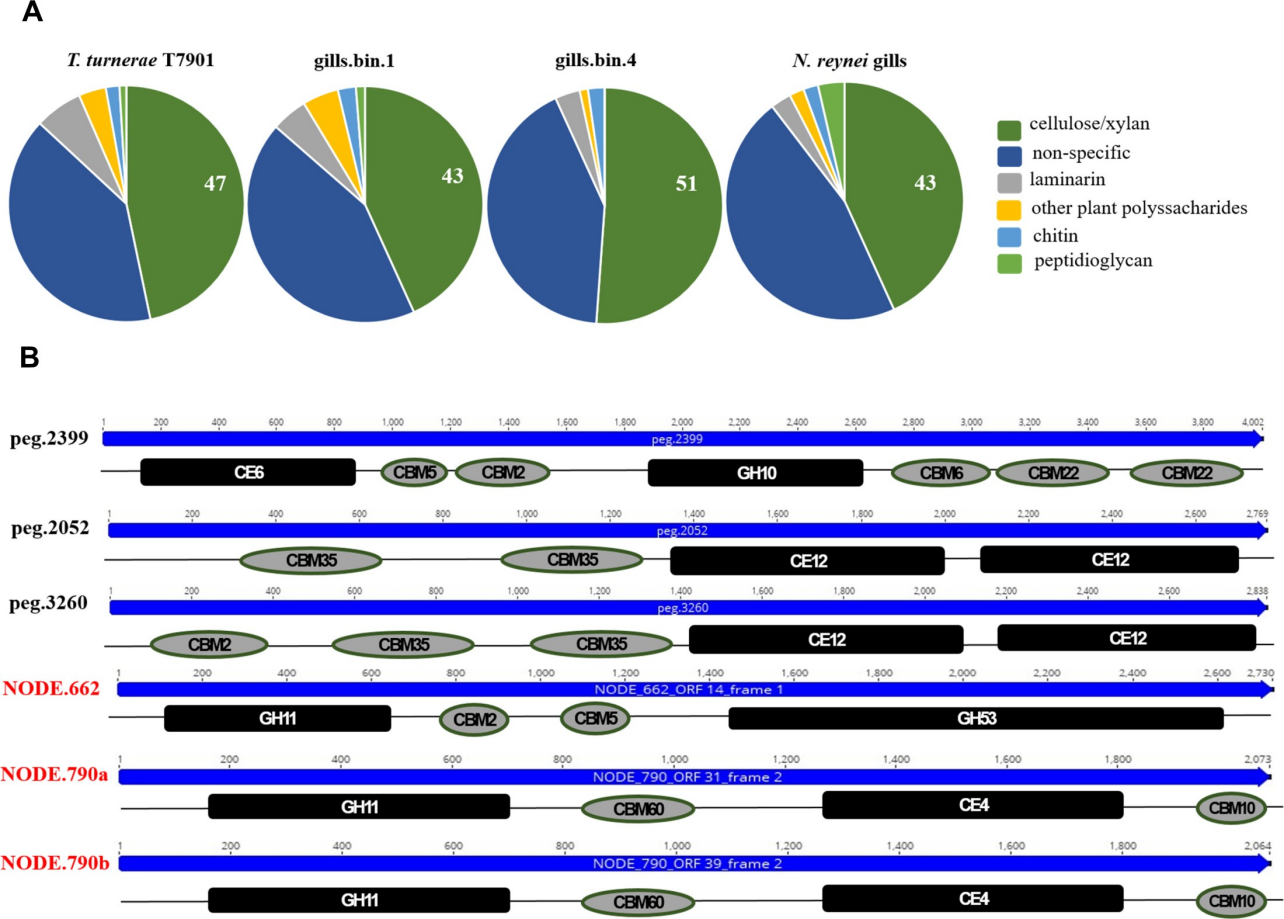
Gill genome bins and un-binned contigs for carbohydrate active enzymes (CAZymes). A) Proportion of GH domains according to the substrate specificity at gills.bin1 and gills.bin 4 binned genomes and at other un-binned contigs from gills metagenome dataset. Substrate specificities as previously defined [9]: dark green = cellulose/xylan GH families (5, 6, 8, 9, 10, 11, 12, 44, 45, 51, 52, 62, and 74); dark blue = GH families with other or not-unique specificities (1, 2, 3, 13, 15, 23, 27, 30, 31, 35, 39, 43, 73, 77, 78, 79, 94, 95, 103, 108, 109, 115, 128, 130); light grey = laminarin GH families (16 and 81); yellow = other plant cell wall polysaccharides GH families (26, 53, and 67); light blue = chitin GH families (18, 19, and 20); light green = peptidoglycan GH families (28 and 105). B) Novel multicatalytic CAZymes configurations detected on *Teredinibacter sp*. gills.bin.4 genome bin (labeled in black), and on un-binned contigs from gill-symbiotic gammaproteobacterial community (labeled in red). Abbreviations key: glycoside hydrolases (GH), carbohydrate esterases (CE), carbohydrate binding modules (CBM). Catalytic domains and binding modules are color coded as black squares and gray circles.

The *T*. *turnerae* T7901 genome codifies an arsenal of enzymes devoted to breaking down lignocellulosic plant material [9]. As such, we annotated the recovered genome bins against the carbohydrate-acting enzymes (CAZy) database [46] and compared their CAZymes profile with the one for T7901, deposited at the CAZy server (S2 Table). Of the three compared genomes, CAZymes represented between 3.5–4% of the protein coding genes, encompassing a diversity of predicted protein domains. Carbohydrate binding modules (CBM) represent between 42–44%, glycoside hydrolases (GH) represent 32–37%, carbohydrate esterases (CE) represent 7–13%, glycoside transferases (GT) represent 9–12%, and polysaccharide lyases (PL) represent 1–2% (S2 Table). Additionally, when GH families were grouped according their substrate specificities, it was shown that the binned genomes also presented the “wood-specialized” profile described for T7901 [9], where a major portion (43–51%) of the GHs are devoted to the digestion of wood-composing polysaccharides (Fig 3A). Furthermore, gills.bin.1 contains identical or near identical (E value = 0.0, amino acids identities ≥ 99%) homologs to the multidomain and multi-catalytic CAZymes detected in the T7901 genome, the exception being TERTU_RS21420, coding for the biochemically characterized multifunctional cellulase CelAB [47] (S3 Table). In comparison, gills.bin.4 codes less conserved homologs (E value = 0.0, amino acids identities ≥ 75%) to only three of T7901 multicatalytic enzymes (S3 Table). However, this genome bin codes for other novel multidomain/multicatalytic enzyme configurations, including a putative acetyl xylan esterase/ endo-1,4-β-xylanase (peg.2399) and two putative dual pectin acetylesterases (peg.2052 and peg.3260) (Fig 3B).

When gills contigs not mapped to the genome bins were mined, additional 291 putative CAZymes were found. Indeed, comparison of CAZy domains relative frequencies, considering the sum of all CAZymes classes (S2 Table), showed that T7901 genome and symbiotic genome bins presented an average enrichment of ~20% of GHs and reduction of ~50% of GTs in comparison to gill metagenome dataset. However, the wood-specialized GH profile is maintained for the entire gill symbiotic community CAZymes (Fig 3A). In fact, other unique multicatalytic lignocellulosic digesting CAZymes that have not binned to *Teredinibacter* genomes were detected, such as a putative endo-β-1,4-xylanase/ endo-β-1,4-galactanase (NODE.622) and two endo-β-1,4-xylanase/acetylxylan esterases (NODE.790a and b) (Fig 3B).

Finally, we evaluate the genome’s CAZymes in light of the recent genome-enabled-proteomic study performed with shipworm specimens of *B*. *setacea* species, which revealed an array of carbohydrate-active-domains derived from the gill symbiotic gammaproteobacterial community are found in the animals’ cecum contents [11]. We verified that, in the compared genomes and in the gill microbiome dataset, the related domain families are enriched, representing 82–89% of the cellulase/xylanases (GH families 5, 6, 9,10, 11, 45 and 53), 50–75% of the carbohydrate esterases (CE families 1, 3, 4, 6 and 15) and 44–62% of the carbohydrate binding motifs detected (CBM2, 10, 22, 57, 60, 61).

In contrast to the lignocellulosic hydrolases present in the gill, contigs from digestive glands and intestine datasets retained only 29 and 101 CAZymes related domains respectively, where GHs are absent in recovered enzymes of digestive glands and account for only 4% of intestine-proteins CAZymes, all of which do not have plant cell wall hydrolysis specificities (S2 Table).

### Biosynthetic gene clusters in the symbiome

**Fig 4:**
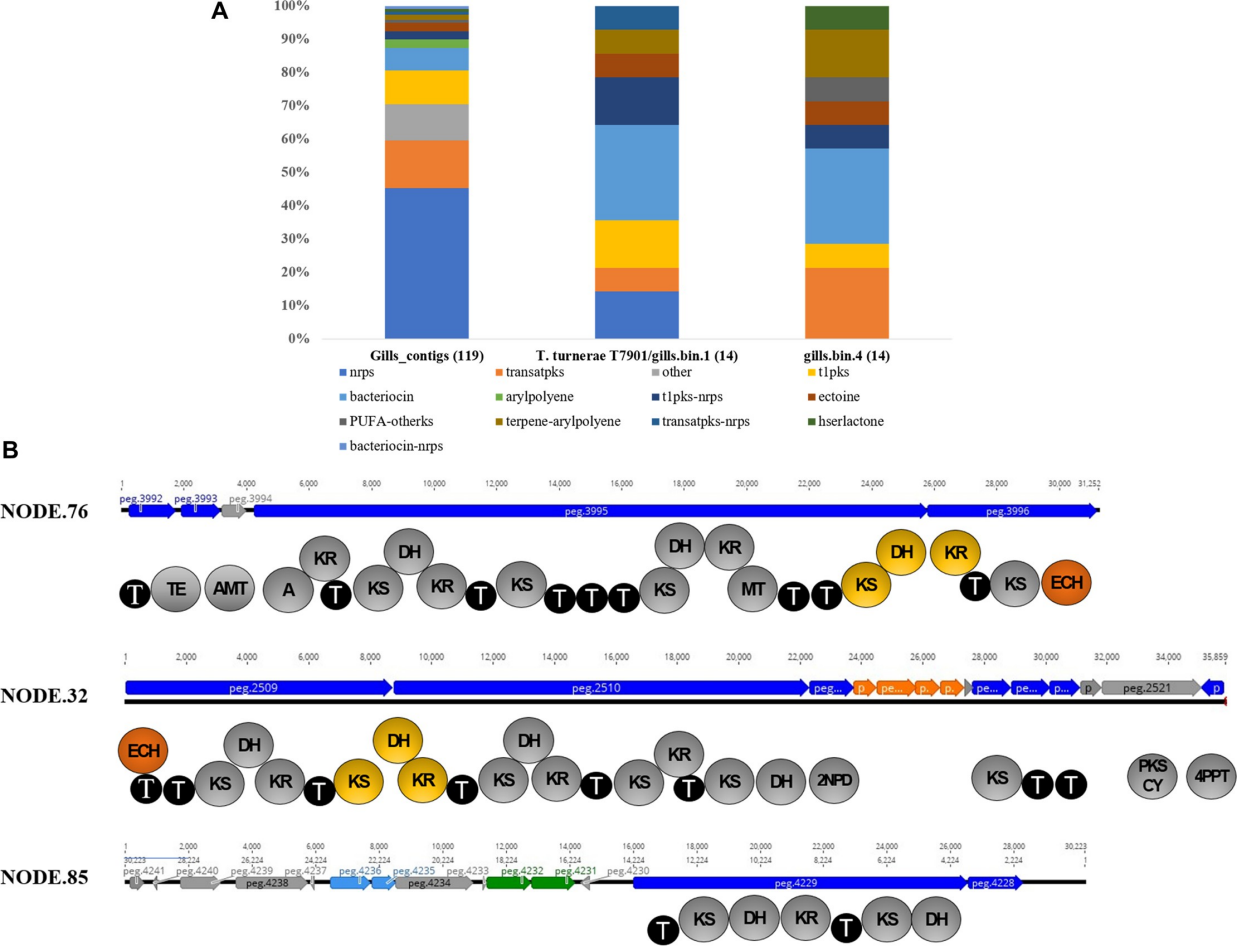
Gill genome bins contigs for Biosynthetic Gene Clusters (BGCs). A) Secondary metabolomes. BGCs/contigs detected in *Teredinibacter turnerae* (T7901 and gills.bin.1), *Teredinibacter sp*.(gills.bin.4) and on contigs derived from *N*. *reynei* gill assembled dataset. Clusters are grouped, and color coded according to the compound class. B) Novel putative *trans*-AT PKS biosynthetic gene clusters detected on *Teredinibacter sp*. gills.bin.4 genome and the main predicted catalytic domains of their open reading frames (ORFs). Biosynthetic, transport-related, regulatory, other β-branching-related genes are color coded in dark-blue, light-blue, green, gray and orange respectively. antiSMASH gene clusters abbreviations (not cited on the main text body): PUFA—Polyunsaturated fatty acids; hrslactone–homoserine lactone. Domains keys: thioesterase (TE), aminotransferase (AMT), adenylation (A), ketosynthase (KS), ketoreductase (KR), dehydrogenase (DH), thiolation (T), enoyl-CoA dehydratases (ECH), 2-Nitropropane dioxygenase (2NPD), polyketide synthase cyclase (PKS CY), 4'-phosphopantetheinyl transferase (4PPT). Putative inter-proteins bimodules are present in yellow, PKS-encoded ECH catalytic domains are present in orange.

Further, we evaluated the potential of the *N*. *reynei* associated microbiome to produce bioactive secondary metabolites. Firstly, the gill, digestive gland and intestine assembled sequence datasets were mined using antiSMASH server [37]. The gill symbiotic microbiome was shown to be a hotspot of BGCs, with a total of 119 contigs encoding enzymes involved in biosynthesis of complex peptides, polyketides and hybrid compounds, besides poly-unsaturated fatty acids (PUFAs), terpenes, arylpolyenes, homoserine lactones, ectoine and bacteriocins (Fig 4A). On the contrary, no secondary metabolite clusters were detected in the digestive gland dataset, and only two contigs for modular type I polyketide synthase (PKS) were found in intestine samples. Intriguingly, the intestine-derived type I PKS contigs best BLASTp hits were to a fusarin C synthetase-like (XP_021367770) and a erythronolide synthase (OWF54747.1) genes linked to the genome of the bivalve *Mizuhopecten yessoensis* (Yesso scallop) [48]. Since modular type I PKS occur almost exclusively in microbes, it is unclear if these homolog genes are truly encoded in the *M*. *yessoensis* genome, which would indicate a rare inter-kingdom horizontal gene transfer event, or result from bacterial DNA contamination of the *M*. *yessoensis* genomic DNA preparation.

We then reanalyzed the secondary metabolome of the reference *T*. *turnerae* T7901 genome using antiSMASH. Previously, T7901 was shown to devote an unexpectedly high percentage (~7%) of its genetic content to natural product biosynthetic pathways [9]. The new genome mining reveals a total of 13 BGCs, adding new putative pathways for bacteriocins (4), terpene (1) and ectoine (1), and condensing firstly delimitated “regions” 3, 4, and 5 [9], into |a putative giant Bacteriocin-Type I PKS (t1pks)-Nrps BGC of ~270.6 kb (S4 Table). Screening of gills.bin.1 and gills.bin.4 genome bins ledto the detection of 19 and 14 contigs, representing putative BGCs, or fragments thereof, for production of compounds from a diversity of classes, such as polyketides, non-ribosomal peptides, terpenes, and bacteriocins (Fig 4A). In addition, we also annotated the secondary metabolome of all genomes in the Cellvibrionaceae family included in our genome-wide phylogeny (Fig 2) and resolved the detected BGCs diversity by supervisioned random forest combined with multidimensional scaling. The *N*. *reynei* gill symbiotic genome bins contain secondary metabolomes highly related to those from representative *T*. *turnerae* strains, which grouped tightly due to enrichments for modular PKS, NRPS and hybrid pathways (S6 Fig).

Eighteen of the putative BGCs from gills.bin.1 and additional fourth contig undetected by the antiSMASH server could be mapped to all 13 BGCs from *T*. *turnerae* T7901 secondary metabolome with high pairwise identity (~99%) and BGCs coverage (~88%) (S4 Table). BLASTp inspection showed that the type 1 PKS route from gills.bin.1 genome bin lacking in T7901 genome is actually conserved in other *T*. *turnerae* genomes, as the ones from strains T8412, T8413 and T8415 (E value = 0, 100% query coverage, ~99% sequence identity) (S5 Table). Therefore, such results corroborate the grouping of gills.1 genome bin as a *T*. *turnerae* strain.

Conversely, only a putative bacteriocin BGC encoded in gills.bin.4 could be mapped to the *T*. *turnerae* T7901 secondary metabolome (S4 Table). BLASTp analysis showed that eight others putative BGCs from this *Teredinibacter* sp. genome bin were also conserved (~97% gene coverage and ~73% identity) in other *Teredinibacter* genomes (S5 Table). The five remaining contigs from gills.bin.4 are novel among the secondary metabolomes of shipworms symbionts.

The core biosynthetic proteins encoded on these BGCs presented best BLASTp hits to putative proteins from Bacteroidetes (~40%), Gammaproteobacteria (33%), and betaproteobacteria (~15%), besides Alphaproteobacteria and Firmicutes (S6 Table). Three of these hypothetical novel BGCs (NODE_76, NODE_26 and NODE_85) code for typical multi-modular *trans*-AT PKS enzymes, with a catalytic domain inventory including two inter-proteins bimodules, commonly involved in formation of α,β-olefinic moieties, and a gene cassette typically involved in the formation of β-branching in bioactive compounds such as the highly cytotoxic pederins and the antibiotic bacillaene [49] (Fig 4B).

### The singularity of *Teredinibacter* resistome within the Cellvibrionaceae family

**Fig 5:**
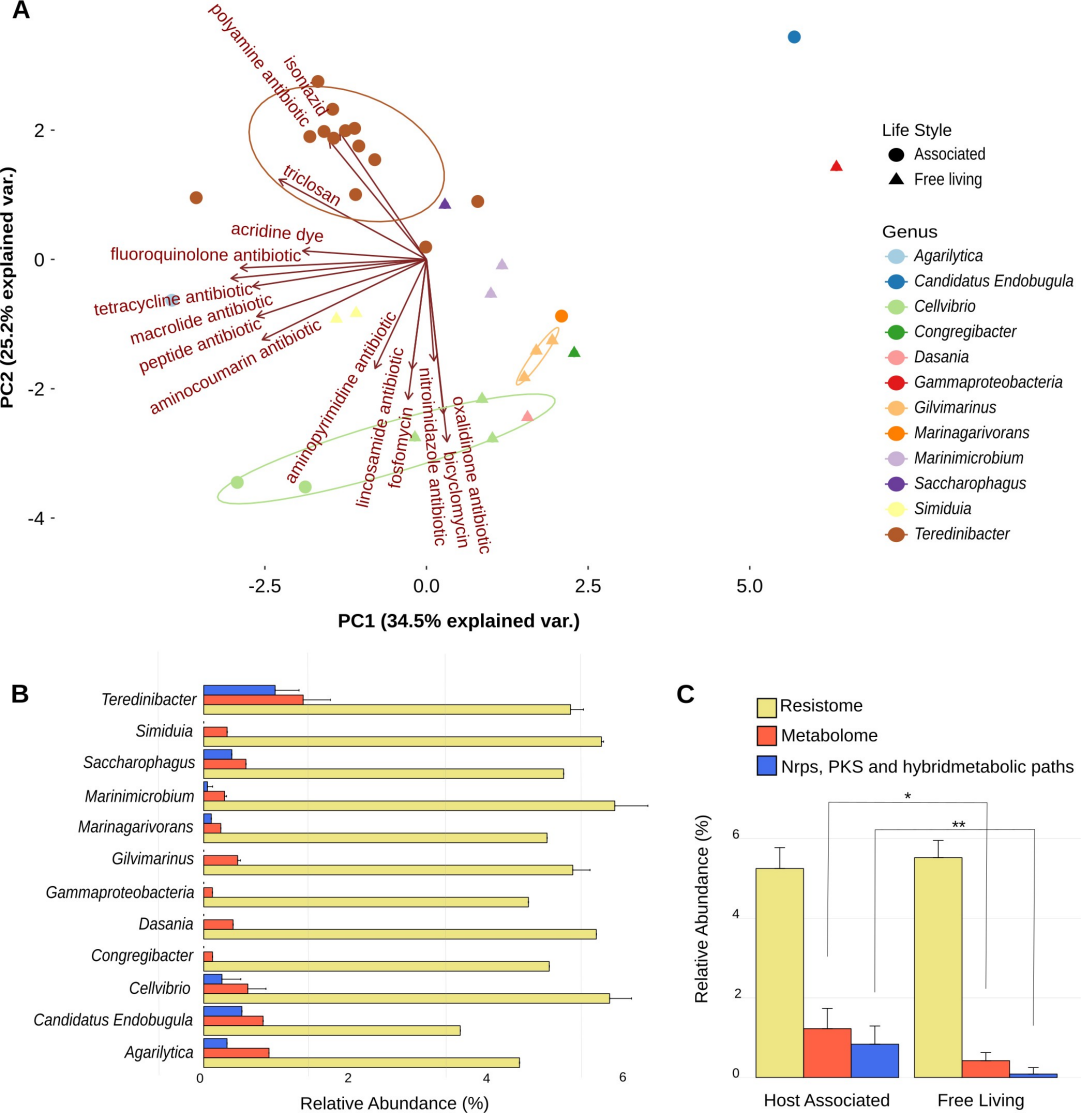
*Teredinibacter* resistome. A) resistome PCA using the fifteen most important genes based on mean decrease accuracy of Random Forest analysis. *Teredinibacter* forms a major group influenced by polyamine, isoniazid, and PK-NRP-derived antibiotics. B) Relative abundance of resistance gene markers, BGCs, and NRPS, PKS and hybrid BGCs. C) Host associated bacteria present a significantly larger metabolome and abundance of NRPS, PKS and their hybrid metabolic paths than free living bacteria. One asterisk represents Kruskal-Wallis test p-values <0.001 and two represent <0.0001 both adjusted with Bonferroni method.

The Resistome is defined as the collection of all antibiotic resistance genes present in a microorganism’s genome, including precursor genes which encode proteins retaining weak antibiotic resistance or binding activity, and that under selective pressure can evolve into a new resistance marker [50]. Here we investigated the resistome coded in *Teredinibacter* genomes, including gills.bin.1 and gills.bin.4, and other representative genomes of the Cellvibrionaceae family. The vast majority of resistance signatures obtained with the Resistance Gene Identifier (RGI) tool from the Comprehensive Antibiotics Resistance Database (CARD) [51] were under the lower stringency cutoffs, which includes putative resistance genes precursors (S7 Table). Multivariate comparisons showed that *Teredinibacter* resistomes grouped tightly and are particularly driven by putative resistance markers to fluoroquinolone (total of 2110 hits) and to antibiotics derived from PKS, NRPS and hybrid compounds, including macrolides (2071), monobactam (2071), tetracycline (1963), penam (1343), peptides (816) and glycopeptide (739) antibiotics (Fig 5A). The relative abundance of genes encoding resistance related proteins were not significantly different (adjusted p-value of 0.28) across the genera inside the Cellvibrionaceae family, suggesting that the resistome size is conserved (Fig 5B, yellow bars). Indeed, resistomes of both *Teredinibacter* (13) and *Cellvibrio* (6) representative genomes were of similar size (representing respectively ~5.3 and ~5.6% of the protein coding genes) (S7 Table). In contrast, BGCs and Nrps-PKS-hybrid metabolic paths relative abundances differed significantly between host-associated and free-living Cellvibrionaceae (adjusted p-values <0.001 and <0.0001, respectively), being larger in the first group (Fig 5B and 5C). Clearly this pattern is driven by symbiotic *Teredinibacter* and, mostly free-living *Cellvibrio* representative genomes (Fig 5B).

## Discussion

In the present work, metagenomics was applied to characterize, compare and investigate the microbiomes associated with the digestive glands, intestines and gills of the mangrove shipworm *N*. *reynei*. Taxonomic annotations showed that[3,4,11] the gills of *N*. *reynei* are a unique symbiotic site, filled with plant cell wall degrading gamma-proteobacteria (Fig 1B and S4 Fig), including bacterial types of the *Teredinibacter* clade (S1B Fig), as seen in other shipworm systems.

In contrast, digestive gland and intestine samples differ from the gill community and are more diverse (Fig 1, S1 and S4 Figs). Such results corroborate previous culture-independent analysis of five shipworms species microbiomes, where digestive tract tissues were reported as virtually sterile (cecum) or containing very low loads of bacteria (intestine) [15].

Therefore, our results indicate that digestive tract microbiomes do not contribute significantly to the digestion of wood particles and consequently the nutrition of the shipworm host. Remarkably, several basal teredinid genera (*Neoteredo*, *Dicyathifer*, *Bactronophorus and Teredothyra*) feature a closed anal canal in which the feces is retained [1,2]. Whilst the morphology of this structure indicates a role in food absorption [18], the microbial community has yet to be explored.

*N*. *reynei* gill metagenomes are enriched for functions under categories of cellular process and signaling (e.g., *cell motility*; *Signal transduction mechanisms*), information storage and processing (e.g., *RNA processing and modification*, *transcription*) and metabolism (e.g., *inorganic ion transport and metabolism*, *Iron acquisition and metabolism*, *Nitrogen metabolism*, and *secondary metabolites biosynthesis*, *transport*, *and catabolism*) (Fig 1D and S2 Fig).

Cross-assemblage based comparative metagenomics showed that such enrichments were related to gammaproteobacterial-derived genes which drive gill samples to group together regardless of specimen location (S3 Fig). In fact, gill-enriched functionalities are consistent with the genetic repertoire described for the *T*. *turnerae*, including nitrogen fixation, iron sequestration and secondary metabolite production [9,10,12].

Specific mining of the contigs assembled from each tissue dataset showed that the *N*. *reynei* gill microbiome is a hot spot of genes for CAZymes, and particularly, wood-degrading hydrolases (Fig 3), and biosynthetic pathways for a variety of bioactive compounds classes (Fig 4). Contrastingly, such a genetic repertoire was shown to be negligible in digestive gland and intestine datasets. However, is important to note that the digestive tract microbiomes are shown to be more diverse than the gill community, therefore higher resolution sequencing is required to fully cover these diverse functionalities.

Two high-quality microbial genomes could be recovered from the gill datasets by our binning strategies (Table 1), and were assigned as *T*. *turnerae* and *Teredinibacter* sp. A phylogeny with several closely related reference taxa confirmed these taxonomical affiliations grouping gills.bin.1 and gills.bin.4 genome bins inside and as a sister group of the *T*. *turnerae* species clade (Fig 2). In addition, the tree topology supports the proposal of new gammaproteobacterial families, including the Cellvibrionaceae family embracing *Teredinibacter* and *Ca*. Endobugula genera of marine invertebrate symbionts [52].

In corroboration with our study, *Teredinibacter* strains were previously isolated from the gills of *Neoteredo reynei* found at the same mangrove site [19]. In addition, other culture independent approaches in *Lyrodus pedicellatus* and *Bankia setacea* have shown that *Teredinibacter* also forms the dominant gill symbiont community [3,4,14].

*Teredinibacter turnerae* populations from a wide variety of shipworm species encompass distinct phylogenetic lineages form two clades (known as Clade I and Clade II) [8]. Our phylogeny reconstitutions using *rpoB* and *gyrB* markers replicate this topology, also agreeing with the genome-wide phylogeny. In both analyses, gills.bin.1 genome bin fell within *T*. *turnerae* “clade I”, just as the *N*. *reynei* isolated *T*. *turnerae* strain T8508 [8,19] (Fig 2 and S5 Fig).

Specific mining revealed that *N*. *reynei*’s symbiotic *Teredinibacter* genome bins contain a repertoire of carbohydrate-active enzymes (CAZymes) that are closely related to the “wood-specialized” profile described in the genome of *T*. *turnerae* [9], which includes a vast repertoire of cellulases/xylanases and other catalytic and binding domains involved in a network for breaking down woody complex polysaccharides (Fig 3). Of particular interest was the detection of multi-catalytic CAZymes with novel domain configurations in gills.bin.4 and unassembled gammaproteobacterial contigs (Fig 3B). An enrichment of catalytic domains were also included within the cabal of CAZymes, which, in the shipworm *B*. *setacea*, have been shown to travel from the symbiotic community located on the gill, to the caecum–the primary site of wood digestion [11]. The well-developed caecum and large anal canal of *N*. *reynei* are features that have been linked to a specialization for wood digestion [17], which is further supported by our discovery of a diverse array of symbiont encoded CAZymes. Therefore, further investigations of *N*. *reynei* symbiotic microbial communities offer significant potential for discovery of biotechnologically relevant wood-digesting CAZymes.

*N*. *reynei* gill symbiotic microbiome was also shown to be a fruitful source of BGCs. Particularly, the *T*. *turnerae* representative genome bin (gills.bin.1) was shown to contain all the BGCs previously described for *T*. *turnerae* strain T7901 [9], including the characterized routes for production of the turnerbactin triscatecholate siderophore [13] and for the tartrolon family of antibiotics [12]. Indeed, these results corroborate the antimicrobial activity previously reported for *T*. *turnerae* strain CS30, isolated from *N*. *reynei* [19], and the results of a PCR survey for the tartrolon producing *trans*-AT PKSs (*trtDEF*) that returned expected amplicons when CS30 and other *T*. *turnerae* isolates genomic DNA were used as template [12]. However, the capacity of *N*. *reynei* symbiotic *T*. *turnerae* to produce tartrolons requires further clarification, since compounds of this family could not be detected on a survey including CS30 strain’s culture chemical extracts [12]. This is of significant interest, as tartrolon antibiotics are thought to play a role on shipworm host chemical defense, and/or gill symbiotic community structuring [12].

Further, the genome bin gills.bin.4 encoded five contigs representing novel clusters among shipworm microbiomes secondary metabolomes (Fig 4, S6 Table). BLASTp searches showed that these clusters putative core biosynthetic proteins present higher similarities to proteins from putative BGCs from Bacteroidetes (as Flavobacteria), Betaproteobacteria (Nesseriales), Alphaproteobacteria (Rhizobiales) and Firmicutes (Bacillus) (S6 Table). As BLAST searches are biased by the database limitation, and that the modular enzymology commonly involved on biosynthesis of secondary metabolites, such as polyketides, contain many conserved catalytic domains and so, are not good taxonomic markers [53], these results may be taken as a suggestion of horizontal gene transfer (HGT) events that led to the acquisition of such pathways by the *Teredinibacter* symbiotic community.

Independently of the acquisition history, the discovery of these novel BGCs reinforces the potential of *N*. *reynei*’s symbiotic system for biotechnological exploration for discovery of new drug leads. In fact, our comparative analyses with representative genomes composing the Cellvibrionaceae family showed that shipworm gammaproteobacterial symbionts, and *Teredinibacter* in particular, share large secondary metabolomes, enriched for PKS-NRPS routes (Fig 5B, S7 Table). This reinforces the hypothesis that production of bioactive metabolites, as polyketides and complex peptides or non-ribosomally derived siderophores increase *Teredinibacter* fitness *in symbio*. Furthermore, considering that each shipworm species has a singular morpho-physiology and each symbiotic gammaproteobacterial community has a unique diversity, specific BGCs might play specific roles within each host-bacteria interaction.

Consequently, the *trans*-AT PSK enzymology detected in the *Teredinibacter* sp. genome bin is significant, since its includes a cassette containing catalytic activities for formation of β-branching chemical structures, present in bryostatins—potent protein kinases modulators, the pederin family of antitumor compounds and myxovirescin antibiotics [49]. Interestingly, macrolide lactones of the bryostatin family, with promising applications for treatment of Alzheimer's disease, had their biosynthesis assigned to a *trans*-AT PKSs cluster (*bry*) from *Ca*. Endobugula sertula, an as yet not cultivated symbiont of the bryozoan *Bugula neritina* [54], also groups within the proposed Cellvibrionaceae family [52]. Therefore, efforts to characterize putative novel trans-AT-PKS systems from *N*. *reynei* isolate strains carrying these clusters are important. In addition, other interesting BGCs were retrieved from the 119 putative BGCs or gene clusters fragments recovered from *N*. *reynei* gill dataset.

Detailed characterization of biosynthetic gene clusters were beyond the scope of this work. However, results presented herein, reaffirm shipworm symbiotic interactions as a prolific source for detection of interesting bioactive compounds, including antibiotics. Indeed, our group and collaborators are working on a deeper and full characterization of the BGC catalog of shipworm symbiotic communities, including isolates genome and gill metagenome samples from several species of shipworms across multiple geographical locations.

The representative *Teredinibacter* genomes also encoded more putative resistance markers for PKS and NRPS derived antibiotics (Fig 5A). The matching of this hypothetical resistome with the profile of *Teredinibacter* BGCs indicate that at least some of these putative markers might truly be resistance to self-produced antibiotics, and so, further characterizations of these genes are of great interest.

## Conclusion

In this study we applied metagenomics to characterize the microbial communities of the digestive gland, intestine and gill of the mangrove specialist shipworm *Neoteredo reynei*. We provided strong evidences that the *N*. *reynei* gill symbiont gammaproteobacterial community is a hot spot for biotechnologically relevant enzymes, such as CAZymes involved on breaking down wood-derived complex polysaccharides, and biosynthetic gene clusters for production of potentially bioactive secondary metabolites such as complex polyketides, peptides and lipopeptides. Further, the discrete bacterial communities forming the microbiome of the digestive tract tissues seems to have little or no involvement in host nutritional support or chemical defense. Digestive gland and intestine datasets presented a simple repertoire of CAZymes, lacking GHs for digestion of woody material, and only one fragmented BGC with hits to a *locus* of the Yesso scallop bivalve mollusk genome. Two representative symbiotic genomes were recovered from gill symbiotic community and taxonomically assigned as a *T*. *turnerae* strain and a yet to be cultivated *Teredinibacter* sp. bacterium. The latter encoded novel multi-catalytic CAZymes, and a *trans*-AT PKS BGC containing the catalytic domains implicated in β-branching in related polyketides. Finally, we demonstrate that the *Teredinibacter* genus is highly enriched in secondary metabolite pathways in comparison to relatives of the Cellvibrionaceae family, including a plethora of BGCs for complex polyketides and nonribosomal peptides, reinforcing the possible role of these compounds on supporting the symbiosis.

## Supporting information

S1 Fig Taxonomic diversity of metagenomes. A) Relative abundance of metagenomes taxonomical signatures under Domain hierarchical level (RefSeq database). B) Relative abundances of metagenomes bacterial genera when considering 16S rRNA reads (RDP database). C) Box-plot of metagenomes Shannon–Weaver index according to the tissue source. Digestive glands (light-blue), and Intestine (green) samples present higher Shannon diversity number, evenness and entropy when compared with Gills (dark-blue) samples. Annotations were performed under MG-RAST server under default stringency parameters (e-value 1e-5, % of identity = 60%, minimal length of 15 and minimal abundance of 1, considering the representative hit).
(TIF)

S2 Fig Two group comparisons between gills and digestive-tract metagenomes annotations. A) Extended error bar plot showing bacterial classes enriched at gills (blue) or digestive tract (digestive glands + intestine, orange) metagenomic groups. B) and C) extended error bar plot showing gills (blue) or digestive tract (orange) enriched bacterial functions when using Cluster of Orthologous (COG) database (B); or Subsystem Technology database (C), at their respective hierarchical level 2. Two groups comparisons were performed at the STAMP software version v2.1.3, as explained at *Methods*.
(TIF)

S3 Fig Comparison of *N*. *reynei* metagenomic samples by cross-assembly. Cladogram representing cross-contigs (i.e., shared contigs containing reads from at least two metagenomes) grouping when using more qualitative distance measures (distance formulas ‘Wootters’ and ‘reads’). Gill samples grouped together regardless the specimen of origin, as highlighted in dark-blue.
(TIF)

S4 Fig Contigs based taxonomical annotation of *N*. *reynei* gills, digestive glands and intestine datasets. A) relative abundances of taxonomical hits per superkingdom. B) Distribution of hits to the most abundant taxon under the phyla taxonomical hierarchy. Inset on gills bar chart brings the distribution of proteobacterial-derived hits (orange bar) as a pizza chart to show the prevalence (98,2%) of hits to the γ-proteobacteria class.
(TIF)

S5 Fig **Neighbor-joining phylogenies inferred for the genome bins protein coding gene markers gyrB (A) and rpoB (B) against a referential dataset of genes from T. turnerae isolates**. The isolates T7901, T7902, T7903, T8401, T8402, T8412, T8415, T8503, T8508, T8509, T8510, T8513, T8601 and T8602 were deposited to the Ocean Genome Legacy Resource (www.oglf.org) and were originally isolated by J. Waterbury (at the collection WHOI) between 1979 and 1986. Isolates obtained from the Philippines Mollusk T0609 and T0611 were obtained from a Lyrodus pedicellatus specimen. Isolates obtained under the project of Philippine Mollusk Symbiont International Cooperative Biodiversity Group (PMS-ICBG) (http://www.pmsicbg.org) have a PMS acronym at their identifications. Bootstrap probability values > 0.7 are shown at the nodes.
(TIF)

S6 Fig Secondary metabolome PCA of Cellvibrionaceae representatives. *N*. *reynei* binned gills-symbiotic genomes gills.bin.1 and gills.bin.4 secondary metabolome grouped closely to a major group formed by *Teredinibacter* and influenced by BGCs for polyketide, non-ribosomal peptide, and hybrid compounds BGCs, besides putative routes for bacteriocins and terpene-arylpolyene.
(TIF)

S1 Table Metagenomes features. * Numbers (1,2,3): N. reynei specimens. Letters (A, B, C): metagenomic samples. ^a^QC–MG-RAST 4.0 applied quality control of the metagenomic paired end reads. ^b^Values obtained after metadata quality control pipeline filtering. ^c^Taxonomical classifications under the Domain hierrchy (RefSeq).
(XLSX)

S2 Table CAZy domains detected on the binned genomes. ND–Not detected.
(DOCX)

S3 Table BLASTp analysis of *T*. *turnerae* T7901 multi-catalytic CAZymes using *N*. *reynei* symbiotic binned genome as database. * T7901 CAZymes for which no highly conserved homolog could be detected on one or both genome bins. Stringency cutoff for highly conserved homologs are E value = 0.0, identity > 75% over the entire query protein.
(DOCX)

S4 Table Binned genome contigs mapped to *Teredinibacter turnerae* T7901 putative Biosynthetic Gene Cluster. * Characterized biosynthetic gene clusters for tartrolon antibiotics and turnerbactin siderophore production. Contigs highlighted in bold were detected by antismash server.
(DOCX)

S5 Table BLASTp analysis showing gills.bin.1 and gills.bin.4 BGCs conserved on *T*. *turnerae* species. (DOCX)

S6 Table BLASTp analysis of the genome bin gills.bin.4 unique BGCs core biosynthetic genes. (DOCX)

S7 Table Cellvibrionaceae representative genome information and resistome and BGCs annotations. * Coding sequence automated annotated by RAST server. † Resistance genes annotated according the CARD server and considering the default stringency cutoffs for perfect/strict and loosely annotations. ‡ Biosynthetic Gene Clusters (BGCs) annotated according to the antiSMASH server, under the bacterial version and using default settings.
(DOCX)
