# Supplementary figures and images for: The gill-associated microbiome is the main source of wood plant polysaccharide hydrolases and secondary metabolite gene clusters in the mangrove shipworm Neoteredo reynei
Source: PLoS One. 2018 Nov 14;13(11):e0200437. doi: 10.1371/journal.pone.0200437 (PMC6235255; doi:10.1371/journal.pone.0200437)

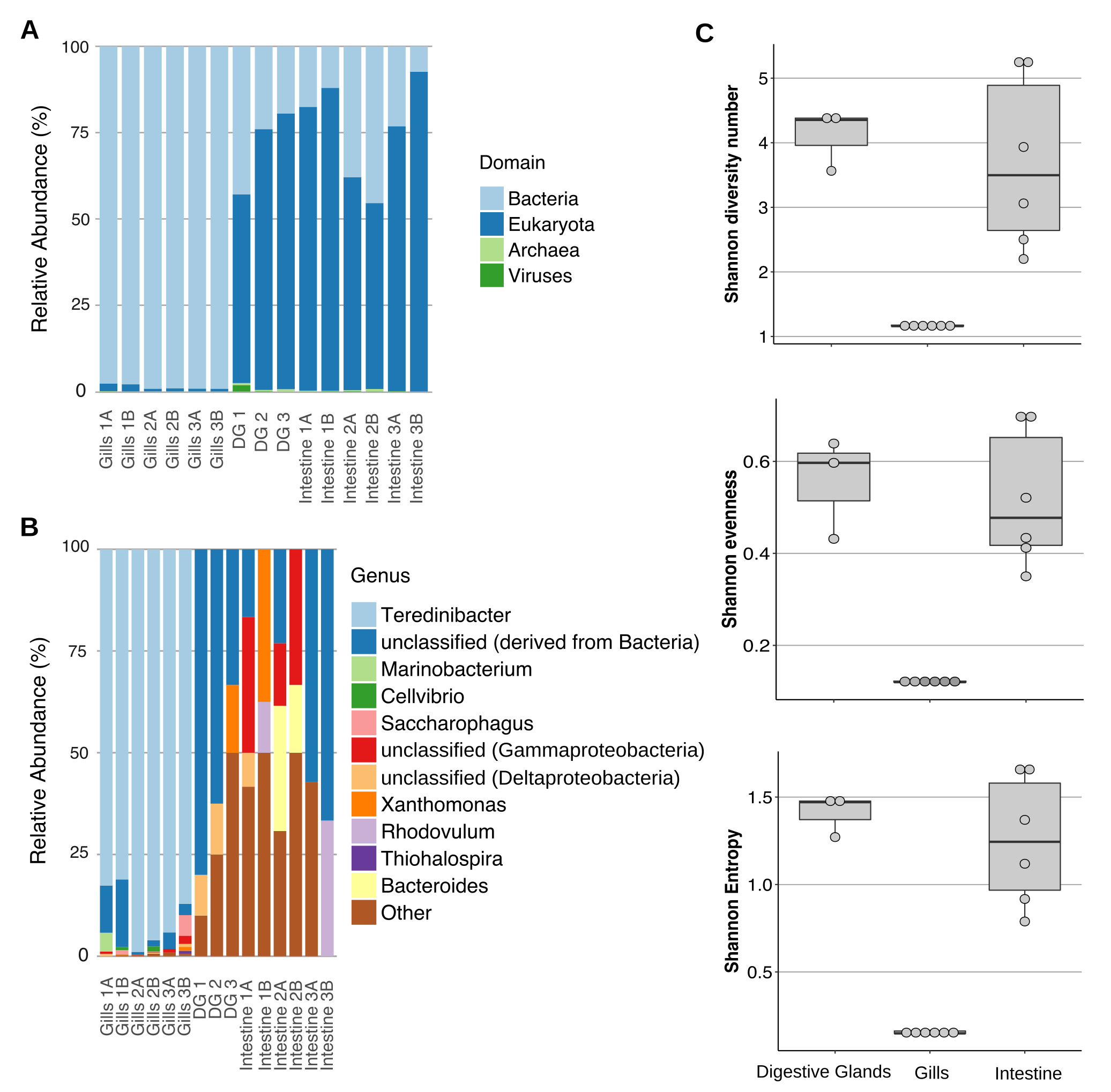

Supplement: S1 Fig — A) Relative abundance of metagenomes taxonomical signatures under Domain hierarchical level (RefSeq database). B) Relative abundances of metagenomes bacterial genera when considering 16S rRNA reads (RDP database). C) Box-plot of metagenomes Shannon–Weaver index according to the tissue source. Digestive glands (light-blue), and Intestine (green) samples present higher Shannon diversity number, evenness and entropy when compared with Gills (dark-blue) samples. Annotations were performed under MG-RAST server under default stringency parameters (e-value 1e-5, % of identity = 60%, minimal length of 15 and minimal abundance of 1, considering the representative hit). (TIF) [file pone.0200437.s001.tif]

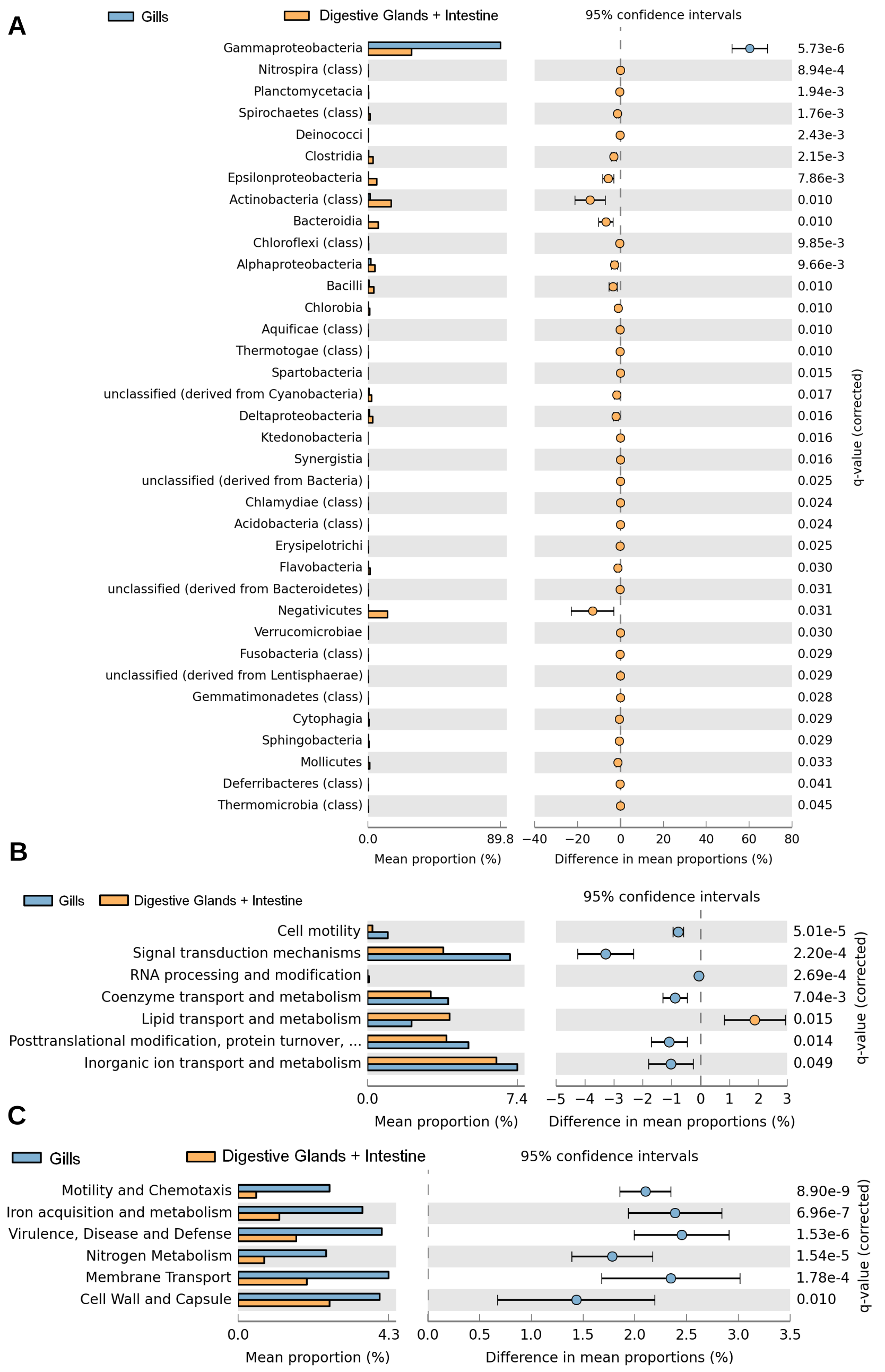

Supplement: S2 Fig — A) Extended error bar plot showing bacterial classes enriched at gills (blue) or digestive tract (digestive glands + intestine, orange) metagenomic groups. B) and C) extended error bar plot showing gills (blue) or digestive tract (orange) enriched bacterial functions when using Cluster of Orthologous (COG) database (B); or Subsystem Technology database (C), at their respective hierarchical level 2. Two groups comparisons were performed at the STAMP software version v2.1.3, as explained at Methods. (TIF) [file pone.0200437.s002.tif]

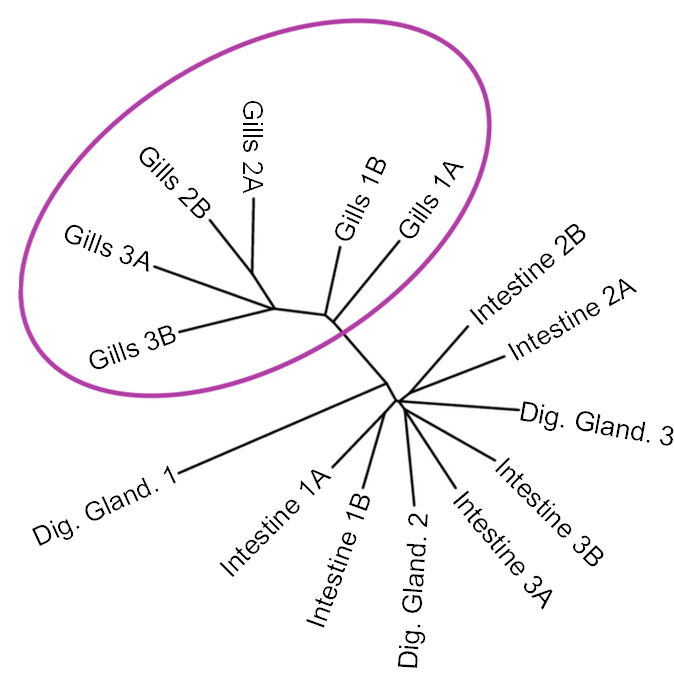

Supplement: S3 Fig — Cladogram representing cross-contigs (i.e., shared contigs containing reads from at least two metagenomes) grouping when using more qualitative distance measures (distance formulas ‘Wootters’ and ‘reads’). Gill samples grouped together regardless the specimen of origin, as highlighted in dark-blue. (TIF) [file pone.0200437.s003.tif]

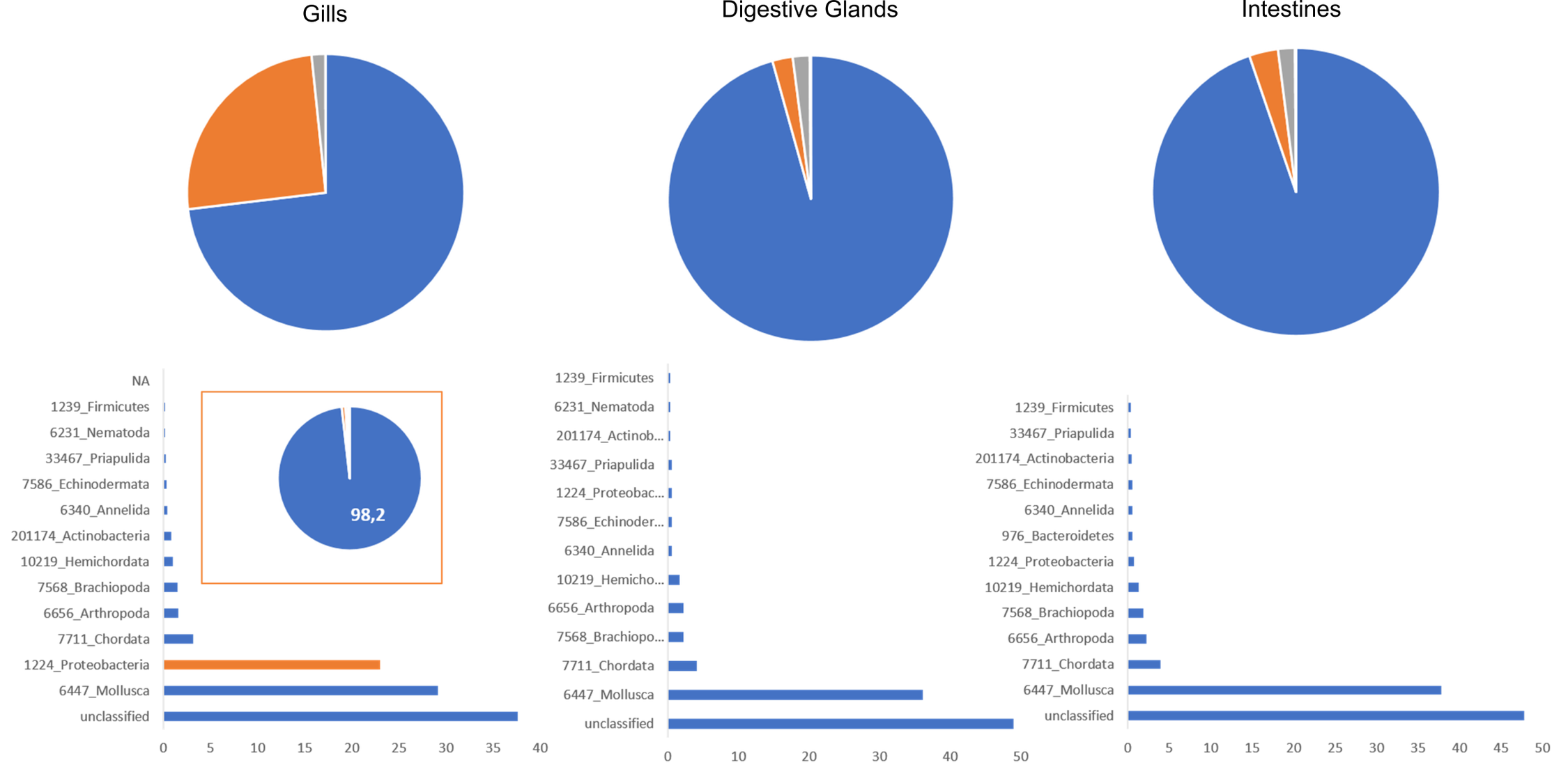

Supplement: S4 Fig — A) relative abundances of taxonomical hits per superkingdom. B) Distribution of hits to the most abundant taxon under the phyla taxonomical hierarchy. Inset on gills bar chart brings the distribution of proteobacterial-derived hits (orange bar) as a pizza chart to show the prevalence (98,2%) of hits to the γ-proteobacteria class. (TIF) [file pone.0200437.s004.tif]

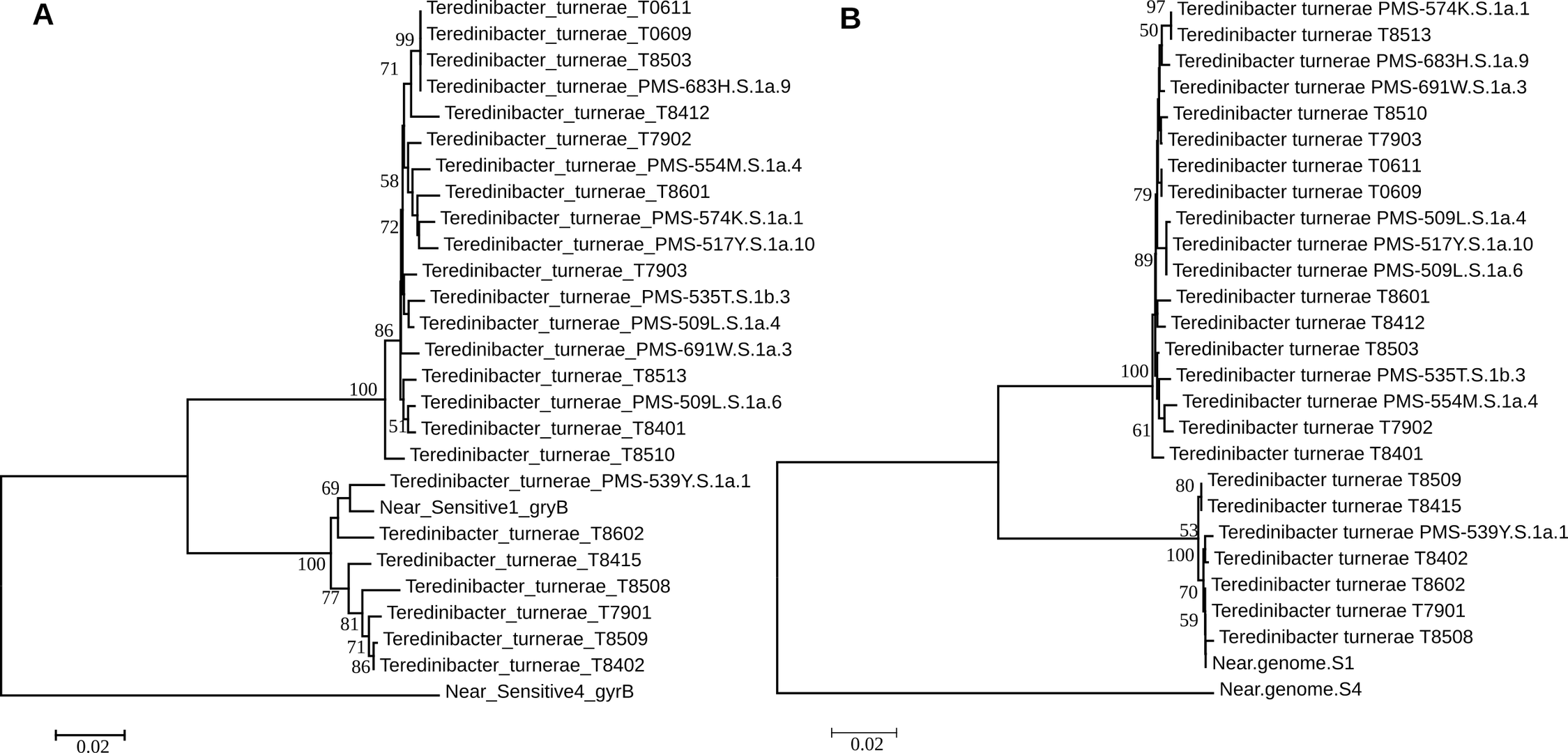

Supplement: S5 Fig — Neighbor-joining phylogenies inferred for the genome bins protein coding gene markers gyrB (A) and rpoB (B) against a referential dataset of genes from T. turnerae isolates. The isolates T7901, T7902, T7903, T8401, T8402, T8412, T8415, T8503, T8508, T8509, T8510, T8513, T8601 and T8602 were deposited to the Ocean Genome Legacy Resource (www.oglf.org) and were originally isolated by J. Waterbury (at the collection WHOI) between 1979 and 1986. Isolates obtained from the Philippines Mollusk T0609 and T0611 were obtained from a Lyrodus pedicellatus specimen. Isolates obtained under the project of Philippine Mollusk Symbiont International Cooperative Biodiversity Group (PMS-ICBG) (http://www.pmsicbg.org) have a PMS acronym at their identifications. Bootstrap probability values > 0.7 are shown at the nodes. (TIF) [file pone.0200437.s005.tif]

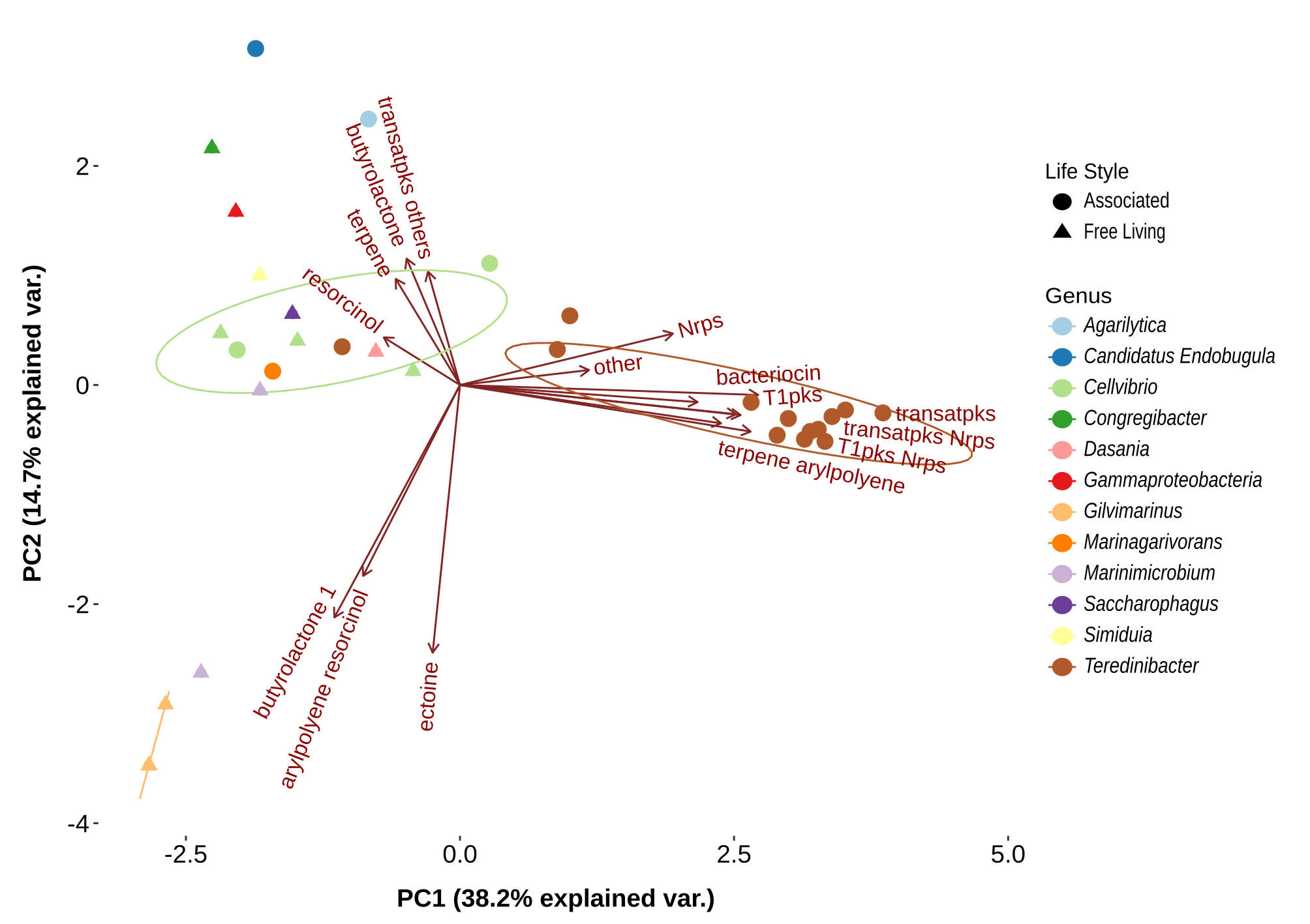

Supplement: S6 Fig — N. reynei binned gills-symbiotic genomes gills.bin.1 and gills.bin.4 secondary metabolome grouped closely to a major group formed by Teredinibacter and influenced by BGCs for polyketide, non-ribosomal peptide, and hybrid compounds BGCs, besides putative routes for bacteriocins and terpene-arylpolyene. (TIF) [file pone.0200437.s006.tif]
