# Supplementary material for: The gill-associated microbiome is the main source of wood plant polysaccharide hydrolases and secondary metabolite gene clusters in the mangrove shipworm Neoteredo reynei
Source: PLoS One. 2018 Nov 14;13(11):e0200437. doi: 10.1371/journal.pone.0200437 (PMC6235255; doi:10.1371/journal.pone.0200437)
Supplement: S2 Table — ND–Not detected. (DOCX) [file pone.0200437.s008.docx]

**Table S2** – CAZy domains detected on the binned genomes.

| Teredinibacter Genomes | T7901 | gills.bin.1 | gills.bin.4 | Gill_contigs | Intestine_contigs | DG_contigs |
| --- | --- | --- | --- | --- | --- | --- |
| CAZymes | 174 | 151 | 163 |  |  |  |
| Glycoside hydrolases (GHs) | 107  (42 families) | 81  (35 families) | 85  (30 families) | 250  (46 families) | 4  (3 families) | ND |
| Carbohydrate esterases (CEs) | 20  (11 families) | 24  (10 families) | 33  (11 families) | 78  (12 families) | 3  (2 families) | 1  (1 families) |
| Polysaccharide lyases (PLs) | 3  (3 families) | 5  (3 families) | 4  (3 families) | 16  (5 families) | 1  (1 family) | ND |
| Glycosyl-transferases (GTs) | 35  (16 families) | 23  (13 families) | 26  (12 families) | 192  (28 families) | 54  (11 families) | 20  (8 families) |
| Carbohydrate binding modules (CBMs) | 120  (16 families) | 106  (19 families) | 120  (21 families) | 358  (30 families) | 48  (10 families) | 9  (2 families) |
| Auxiliary activities (AAs) | 1  (1 families) | 4  (3 families) | 3  (3 families) | 10  (5 families) | ND | ND |

ND – Not detected
