# Supplementary material for: The gill-associated microbiome is the main source of wood plant polysaccharide hydrolases and secondary metabolite gene clusters in the mangrove shipworm Neoteredo reynei
Source: PLoS One. 2018 Nov 14;13(11):e0200437. doi: 10.1371/journal.pone.0200437 (PMC6235255; doi:10.1371/journal.pone.0200437)
Supplement: S3 Table — * T7901 CAZymes for which no highly conserved homolog could be detected on one or both genome bins. Stringency cutoff for highly conserved homologs are E value = 0.0, identity > 75% over the entire query protein. (DOCX) [file pone.0200437.s009.docx]

**Table S3 – BLASTp analysis of *T. turnerae* T7901 multi-catalytic CAZymes using *N. reynei* symbiotic binned genomes as database.**

| T7901 Cazymes  (size in aa) | gills.bin.1 Identical/Homolog protein (size in aa) | E value | identities (%) | Positivities (%) | Gaps (%) |
| --- | --- | --- | --- | --- | --- |
| TERTU_RS01885 (894) | 2426.6.peg.600 (894) | 0.0 | 892/894 (99%) | 894/894 (100%) | no |
| TERTU_RS06560 (1276) | 2426.6.peg.3052 (1262) | 0.0 | 1253/1262 (99%) | 1257/1262 (99%) | no |
| TERTU_RS07445 (687) | 2426.6.peg.3206 | 0.0 | 685/687 (99%) | 685/687 (99%) | no |
| TERTU_RS07450 (685) | 2426.6.peg.3207 | 0.0 | 684/685 (99%) | 685/685 (100%) | no |
| TERTU_RS21420 (1010)* | 2426.6.peg.1591 | e-163, | 220/302 (72%) | 257/302 (85%) | no |
| TERTU_RS15260 (1051) | 2426.6.peg.105 (1051) | 0.0 | 1050/1051 (99%) | 1051/1051 (100%) | no |
| TERTU_RS15970 (952) | 2426.6.peg.922 (952) | 0.0 | 952/952 (100%) | 952/952 (100%) | no |
| T7901 Cazymes  (size in aa) | **gills.bin.4 Identical/Homolog protein (size in aa)** | **E value** | **identities (%)** | **Positivities (%)** | **Gaps (%)** |
| TERTU_RS01885 (894) | 2426.4.peg.2965 (913) | 0.0 | 749/927 (80% | 808/927 (87%) | 47/927 (5%) |
| TERTU_RS06560 (1276)* | 2426.4.peg.4238 (750) | e-170 | 298/522 (57%) | 387/522 (74%) | 8/522 (1%) |
| TERTU_RS07445 (687) | 2426.4.peg.862 (685) | 0.0 | 528/690 (76%) | 588/690 (85%) | 8/690 (1%) |
| TERTU_RS07450 (685) | 2426.4.peg.863 (674) | 0.0 | 560/685 (81%) | 608/685 (88%) | 11/685 (1%) |
| TERTU_RS21420 (1010)* | 2426.4.peg.702 (647) | 0.0 | 421/526 (80%) | 471/526 (89%) | 5/526 (0%) |
| TERTU_RS15260 (1051) | 2426.4.peg.4014 (1069) | 0.0 | 852/1050 (81%) | 922/1050 (87%) | 20/1050 (1%) |
| TERTU_RS15970 (952)* | 2426.4.peg.2400 (498) | 0.0 | 431/501 (86%) | 455/501 (90%) | 10/501 (1%) |

*T7901 CAZymes for which no highly conserved homolog could be detected on one or both genome bins. Stringency cutoff for highly conserved homologs are E value = 0.0, identity > 75% over the entire query protein.
