# Supplementary material for: The gill-associated microbiome is the main source of wood plant polysaccharide hydrolases and secondary metabolite gene clusters in the mangrove shipworm Neoteredo reynei
Source: PLoS One. 2018 Nov 14;13(11):e0200437. doi: 10.1371/journal.pone.0200437 (PMC6235255; doi:10.1371/journal.pone.0200437)
Supplement: S4 Table — * Characterized biosynthetic gene clusters for tartrolon antibiotics and turnerbactin siderophore production. Contigs highlighted in bold were detected by antismash server. (DOCX) [file pone.0200437.s010.docx]

**Table S4 – Genome bins contigs mapped to *Teredinibacter turnerae* T7901 putautive Biosynthetic Gene Cluster.**

| *Teredinibacter turnerae* T7901  BGCs (class) | Genomic locus (From/to) | gills.bin.1 mapped  contigs (bp) | Pairwise  Identity | Referential  BGC coverage |
| --- | --- | --- | --- | --- |
| Cluster 1  (Bacteriocin) | 997220/1008065 | NODE_21 (59.511); NODE_455 (7.649**)** | 100% | 84% |
| Cluster 2  (Bacteriocin) | 1599594/1609812 | *NODE_154 (19.342)*; NODE_233 (14.348) | 99% | 89% |
| Cluster 3  (Transatpks-Nrps) | 2015079/2099533 | *NODE_6 (97.957)*; NODE_4272 (3.449);  NODE_390 (8.846); NODE_320 (10.648) | 99% | 96% |
| *Cluster 4  (Tartrolon_BGC) | 2266203/2348742 | *NODE_170 (18,099)*; NODE_851 (5,509);  NODE_533 (6,855); NODE_958 (5,263);  NODE_2565 (3912); NODE_600 (6462) | 98% | 92% |
| Cluster 5  (Bacteriocin-T1pks-Nrps) | 2405094/2675765 | NODE_665 (6.172); *NODE_179 (17.521)*;  NODE_1138 (4.966); *NODE_160 (19.146)*;  NODE_6499 (3.089); NODE_582 (6.539);  NODE_1051 (5.104); *NODE_47 (40.121)*;  *NODE_420 (8.184)*; *NODE_9930 (2.743)*;  NODE_1490 (4.568); NODE_141 (20.439);  NODE_486 (7.288); *NODE_282 (12.535)* | 97% | 59% |
| Cluster 6  (Nrps) | 3137224/3218534 | NODE_194 (16377); NODE_9275 (2.796);  *NODE_307 (11.076)*; *NODE_133 (22.294)*;  NODE_202 (15.995); NODE_9507 (2.777);  NODE_410 (8.342) | 96% | 86% |
| Cluster 7  (Terpene-Arylpolyene) | 3570053/3657097 | NODE_285 (12.231); NODE_681 (6.060);  NODE_376 (9.223); *NODE_126 (23.232)*;  *NODE_59 (36.772)*; NODE_707 (5.957);  NODE_431 (7.977) | 99% | 91% |
| Cluster 8  (Ectoine) | 3742732/3753130 | *NODE_283 (12.442)* | 100% | 100% |
| Cluster 9  (Bacteriocin) | 3902284/3913171 | NODE_277 (12.604); NODE_11728 (2.612) | 99% | 72% |
| *Cluster 10  (Turnerbactin_BGC) | 4469354/4525734 | NODE_230 (14,691); *NODE_129 (22,831)* | 99% | 100% |
| Cluster 11  (T1pks-Nrps) | 4716461/4768285 | *NODE_163 (18,957)*; NODE_9703 (2,761);  NODE_448 (7,725); NODE_1505 (4,551),  NODE_31 (50783) | 99% | 79% |
| Cluster 12  (T1pks) | 4843659/4890084 | NODE_9703 (2.761); NODE_448 (7.725);  *NODE_163 (18.957)*; NODE_1505 (4.551);  NODE_31 (50.783) | 99% | 93% |
| Cluster 13  (Bacteriocin) | 50712125082192 | *NODE_207 (15.486)* | 99% | 100% |
| Average |  |  | 99% | 88% |
| *Teredinibacter turnerae* T7901  BGCs (class) | **Genomic locus (From/to)** | **gills.bin.4**  **mapped contig (bp)** | **Pairwise**  **Identity** | **Referential**  **BGC coverage** |
|  |  |  |  |  |
| Cluster 1 (Bacteriocin) | 997220/1008065 | NODE_3991 (3.511) | 83% | 32% |
| Cluster 6 (Nrps) | 3137224/3218534 | NODE_590 (6.509) | 70% | 12% |
| Cluster 9 (Bacteriocin) | 3902284/3913171 | *NODE_137 (20,843)*; NODE_144 (20,122);  NODE_629 (6,335) | 92% | 52% |
| Average |  |  | 81% | 32% |

*Characterized biosynthetic gene clusters for tartrolon antibiotics and turnerbactin siderophore production.

Contigs highlighted in italics and underline were detected by antismash server.
