# Supplementary material for: The gill-associated microbiome is the main source of wood plant polysaccharide hydrolases and secondary metabolite gene clusters in the mangrove shipworm Neoteredo reynei
Source: PLoS One. 2018 Nov 14;13(11):e0200437. doi: 10.1371/journal.pone.0200437 (PMC6235255; doi:10.1371/journal.pone.0200437)
Supplement: S5 Table — (DOCX) [file pone.0200437.s011.docx]

**Table S5: BLASTp analysis showing gills.bin.1 and gills.bin.4 BGCs conserved on *T. turnerae* species**

| Binned Genome | antismash  detected BGC | antiSMASH Putaitive BGC Proteins | Biggest  ptn homology | Organism | Query cover | E value | Identity |
| --- | --- | --- | --- | --- | --- | --- | --- |
| gills.bin.1 | **Cluster 17 - T1pks** | no_tag_f_3437 | WP_019606253.1 [hypothetical protein] | Teredinibacter turnerae | 100% | 0 | 98% |
|  |  | no_tag_f_3439 | WP_019601469.1 [N-succinylglutamate 5-semialdehyde dehydrogenase] | Teredinibacter turnerae | 100% | 0 | 99% |
|  |  | no_tag_f_3448 | WP_028886585.1 [thioesterase] | Teredinibacter turnerae | 100% | 0 | 98% |
|  |  | no_tag_f_3450 | WP_018417065.1 [hypothetical protein] | Teredinibacter turnerae | 100% | 0 | 100% |
|  |  | no_tag_f_3452 | WP_018276995.1 [MULTISPECIES: hypothetical protein] | Teredinibacter | 100% | 8,00E-47 | 100% |
|  |  | no_tag_f_3453 | WP_019601479.1 [hypothetical protein] | Teredinibacter turnerae | 100% | 0 | 98% |
|  |  | no_tag_f_3454 | WP_028877620.1 [3-oxoacyl-ACP synthase] | Teredinibacter turnerae | 100% | 0 | 99% |
|  |  | no_tag_f_3455 | WP_018014694.1 [type I polyketide synthase] | Teredinibacter turnerae | 100% | 0 | 99% |
|  |  | no_tag_f_3456 | WP_028877622.1 [polyketide synthase] | Teredinibacter turnerae | 100% | 0 | 99% |
|  |  | no_tag_f_3457 | WP_028877623.1 [polyketide synthase] | Teredinibacter turnerae | 100% | 0 | 97% |
| gills.bin.4 | **Cluster 4 - Bacteriocin (17154)** | no_tag_f_1432 | WP_018014281 [DUF692 domain-containing protein] | Teredinibacter turnerae | 86% | 1,00E-116 | 61% |
|  |  | no_tag_f_1435 | WP_019602057 [hypothetical protein] | Teredinibacter turnerae | 100% | 7,00E-102 | 81% |
|  | **Cluster 7 - Terpene (23366)** | no_tag_f_2177 | WP_019606292 [(2E,6E)-farnesyl diphosphate synthase] | Teredinibacter turnerae | 100% | 4,00E-144 | 71% |
|  |  | no_tag_f_2178 | WP_018274589 [MULTISPECIES: hydroxymethylglutaryl-CoA synthase] | Teredinibacter | 100% | 0 | 89% |
|  |  | no_tag_f_2179 | WP_019602221 [hopanoid biosynthesis associated radical SAM protein HpnH] | Teredinibacter turnerae | 100% | 0 | 84% |
|  |  | no_tag_f_2182 | WP_028883164 [hydroxymethylglutaryl-CoA reductase, degradative] | Teredinibacter turnerae | 100% | 0 | 69% |
|  |  | no_tag_f_2185 | WP_018414840 [NAD-dependent dehydratase] | Teredinibacter turnerae | 93% | 4,00E-130 | 71% |
|  |  | no_tag_f_2188 | WP_019606286 [MULTISPECIES: phytoene/squalene synthase family protein] | Teredinibacter | 97% | 0 | 78% |
|  |  | no_tag_f_2189 | WP_019606285 [squalene-hopene cyclase] | Teredinibacter turnerae | 98% | 0 | 70% |
|  |  | no_tag_f_2192 | ACR13269 [putative membrane protein] | Teredinibacter turnerae T7901 | 99% | 0 | 55% |
|  |  | no_tag_f_2198 | WP_028883147 [GGDEF domain-containing protein] | Teredinibacter turnerae | 100% | 1,00E-75 | 43% |
|  | **Cluster 8 - Bacteriocin (5708)** | no_tag_f_2321 | WP_028886007 [DUF692 domain-containing protein] | Teredinibacter turnerae | 100% | 2,00E-169 | 99% |
|  | **Cluster 10 - Bacteriocin (10923)** | no_tag_f_3419 | WP_015817799 [octaprenyl diphosphate synthase] | Teredinibacter turnerae | 99% | 0 | 87% |
|  |  | no_tag_f_3422 | WP_075185962 [DUF692 domain-containing protein] | Alteromonadales bacterium BS08 | 90% | 1,00E-128 | 61% |
|  |  | no_tag_f_3425 | WP_018274044 [MULTISPECIES: UTP--glucose-1-phosphate uridylyltransferase ] | Teredinibacter | 100% | 0 | 95% |
|  |  | no_tag_f_3426 | WP_085158791 [3-hydroxyacyl-[acyl-carrier-protein]] | Alteromonadaceae bacterium Bs31 | 97% | 2,00E-113 | 92% |
|  |  | no_tag_f_3427 | WP_015818656 [beta-ketoacyl-[acyl-carrier-protein] synthase I] | Teredinibacter turnerae | 100% | 0 | 89% |
|  | **Cluster 11 - T1pks-nrps (41682)** | no_tag_f_3556 | WP_019601126 [asparagine synthetase B] | Teredinibacter turnerae | 100% | 0 | 75% |
|  |  | no_tag_f_3557 | WP_019605180 [hypothetical protein] | Teredinibacter turnerae | 100% | 2,00E-40 | 82% |
|  |  | no_tag_f_3558 | WP_015818645 [carbohydrate esterase family 1 domain-containing protein] | Teredinibacter turnerae | 88% | 7,00E-116 | 64% |
|  |  | no_tag_f_3559 | WP_026193645 [MULTISPECIES: polyketide synthase] | Teredinibacter | 98% | 0 | 72% |
|  |  | no_tag_f_3560 | WP_028881172 [acyltransferase] | Teredinibacter turnerae | 99% | 8,00E-146 | 64% |
|  |  | no_tag_f_3562 | WP_028876624 [non-ribosomal peptide synthetase] | Teredinibacter turnerae | 99% | 0 | 61% |
|  |  | no_tag_f_3563 | WP_018416509 [hypothetical protein] | Teredinibacter turnerae | 99% | 1,00E-149 | 60% |
|  |  | no_tag_f_3564 | WP_015816858 [condensation domain-containing protein] | Teredinibacter turnerae | 99% | 5,00E-163 | 49% |
|  |  | no_tag_f_3571 | WP_045826724 [short-chain dehydrogenase] | Teredinibacter sp. 991H.S.0a.06 | 100% | 5,00E-142 | 76% |
|  | **Cluster 12 - T1pks (46406)** | no_tag_f_3737 | WP_018015359 [type I polyketide synthase] | Teredinibacter turnerae | 99% | 0 | 75% |
|  |  | no_tag_f_3738 | WP_015817914 [PaaI family thioesterase] | Teredinibacter turnerae | 100% | 7,00E-98 | 93% |
|  |  | no_tag_f_3740 | WP_015818520 [thioesterase] | Teredinibacter turnerae | 97% | 3,00E-107 | 83% |
|  |  | no_tag_f_3742 | WP_028887095 [hypothetical protein] | Teredinibacter turnerae | 99% | 6,00E-179 | 68% |
|  |  | no_tag_f_3743 | WP_018416318 [hypothetical protein] | Teredinibacter turnerae | 96% | 0 | 63% |
|  |  | no_tag_f_3753 | WP_018416533 [glutamine--fructose-6-phosphate transaminase (isomerizing)] | Teredinibacter turnerae | 100% | 0 | 87% |
|  |  | no_tag_f_3755 | WP_028876596 [GGDEF domain-containing protein] | Teredinibacter turnerae | 99% | 0 | 65% |
|  | **Cluster 13 - Bacteriocin (9952)** | no_tag_f_4006 | WP_045825326 [glutathione S-transferase] | Teredinibacter sp. 991H.S.0a.06 | 99% | 3,00E-102 | 68% |
|  |  | no_tag_f_4009 | WP_015819303 [Nif11-like leader peptide family natural product precursor] | Teredinibacter turnerae | 100% | 6,00E-37 | 85% |
|  | **Cluster 14 - Arylpolyene (25950)** | no_tag_f_4223 | WP_015817095 [23S rRNA (uridine(2552)-2'-O)-methyltransferase RlmE] | Teredinibacter turnerae | 92% | 2,00E-128 | 85% |
|  |  | no_tag_f_4231 | WP_028883175 [beta-ketoacyl-[acyl-carrier-protein] synthase II] | Teredinibacter turnerae | 100% | 0 | 70% |
|  |  | no_tag_f_4233 | WP_015817095 [23S rRNA (uridine(2552)-2'-O)-methyltransferase RlmE] | Teredinibacter turnerae | 58% | 1,00E-18 | 65% |
|  |  | no_tag_f_4234 | WP_045826208 [hypothetical protein] | Teredinibacter sp. 991H.S.0a.06 | 99% | 0 | 63% |
|  |  | no_tag_f_4237 | WP_018274614 [MULTISPECIES: ubiquinone biosynthesis protein UbiE] | Teredinibacter | 100% | 8,00E-167 | 90% |
|  |  | no_tag_f_4239 | WP_018274616 [MULTISPECIES: 1-acyl-sn-glycerol-3-phosphate acyltransferase] | Teredinibacter | 100% | 5,00E-148 | 77% |
|  |  | no_tag_f_4240 | WP_015819732 [acyl carrier protein] | Teredinibacter turnerae | 100% | 4,00E-47 | 83% |
|  |  | no_tag_f_4242 | WP_018014085 [glycosyltransferase family 2 protein] | Teredinibacter turnerae | 100% | 8,00E-145 | 79% |
|  |  | no_tag_f_4245 | WP_015817910 [hypothetical protein] | Teredinibacter turnerae | 98% | 0 | 53% |
|  |  | no_tag_f_4247 | WP_018414816 [MULTISPECIES: 3-oxoacyl-ACP reductase FabG] | Teredinibacter | 100% | 2,00E-147 | 86% |
|  |  | no_tag_f_4248 | WP_026337509 [beta-ketoacyl-ACP synthase II] | Teredinibacter turnerae | 100% | 0 | 85% |
