# Supplementary material for: The gill-associated microbiome is the main source of wood plant polysaccharide hydrolases and secondary metabolite gene clusters in the mangrove shipworm Neoteredo reynei
Source: PLoS One. 2018 Nov 14;13(11):e0200437. doi: 10.1371/journal.pone.0200437 (PMC6235255; doi:10.1371/journal.pone.0200437)
Supplement: S6 Table — (DOCX) [file pone.0200437.s012.docx]

**Table S6: BLASTp analysis of the genome bin gills.bin.4 unique BGCs core biosynthetic genes.**

| BGC Class  (size pb) | Biosynthetic Ptn | Homologous ptn  accession number [function] | Referential  Organism | Query cover | E value | Identity |
| --- | --- | --- | --- | --- | --- | --- |
| Cluster 1 - Hserlactone (12,070) | no_tag_f_92 | WP_073631649.1 [oxidoreductase] | Pseudoxanthobacter soli | 97% | 3,00E-125 | 66% |
|  | no_tag_f_105 | WP_045857553 [autoinducer synthesis protein] | Alteromonadaceae bacterium Bs12 | 96% | 3,00E-84 | 60% |
| Cluster 2 - Transatpks (31252) | no_tag_f_807 | WP_074405474 [hypothetical protein] | Aquimarina megaterium | 99% | 3,00E-70 | 30% |
|  | no_tag_f_808 | WP_046744425 [GMC family oxidoreductase] | Kordia zhangzhouensis | 98% | 5,00E-127 | 45% |
|  | no_tag_f_810 | WP_087684108.1 [hypothetical protein] | Agarilytica rhodophyticola | 0,76 | 0 | 37% |
|  | no_tag_f_811 | WP_009579370 [malonyl CoA-ACP transacylase] | Fulvivirga imtechensis | 96% | 0 | 43% |
| Cluster 3 - Otherks-Pufa (46357) | no_tag_f_1450 | WP_018416436 [glutathione-dependent disulfide-bond oxidoreductase] | Teredinibacter turnerae | 0,99 | 4E-154 | 0,74 |
|  | no_tag_f_1453 | WP_075186081 [PLP-dependent aminotransferase family protein] | Alteromonadales bacterium BS08 | 95% | 6,00E-147 | 57% |
|  | no_tag_f_1457 | WP_043641638 [type I polyketide synthase] | Chromobacterium haemolyticum | 99% | 0 | 45% |
|  | no_tag_f_1458 | WP_081544595 [beta-ketoacyl synthase] | Chromobacterium haemolyticum | 99% | 0 | 50% |
|  | no_tag_f_1459 | WP_082151102 [PfaD family protein] | Chromobacterium sp. LK1 | 100% | 0 | 60% |
|  | no_tag_f_1460 | WP_038243220 [3-oxoacyl-ACP reductase] | Xenorhabdus bovienii | 100% | 1,00E-108 | 62% |
|  | no_tag_f_1461 | WP_048410695 [thioester reductase] | Chromobacterium sp. LK1 | 97% | 2,00E-147 | 51% |
|  | no_tag_f_1462 | WP_077982302 [amidohydrolase] | Agrobacterium salinitolerans | 97% | 4,00E-89 | 51% |
|  | no_tag_f_1932 | WP_090298852 [glycosyl hydrolase] | Muricauda zhangzhouensis | 70% | 0 | 63% |
| Cluster 6 - Transatpks (30223) | no_tag_f_2042 | WP_074405475 [hypothetical protein] | Aquimarina megaterium | 91% | 0 | 60% |
|  | no_tag_f_2043 | WP_034246254 [hypothetical protein] | Aquimarina atlantica | 99% | 0 | 50% |
| Cluster 9 - Transatpks-Otherks | no_tag_f_2726 | WP_009579370 [malonyl CoA-ACP transacylase] | Fulvivirga imtechensis | 98% | 0 | 43% |
|  | no_tag_f_2727 | WP_024772258 [hypothetical protein] | Aquimarina macrocephali | 99% | 0 | 44% |
|  | no_tag_f_2728 | WP_081310687 [hypothetical protein] | Pseudoalteromonas luteoviolacea | 94% | 0 | 56% |
|  | no_tag_f_2729 | WP_077412984 [MBL fold metallo-hydrolase] | Marinicella sp. F2 | 94% | 9,00E-85 | 52% |
|  | no_tag_f_2730 | WP_065792709 [3-hydroxy-3-methylglutaryl-ACP synthase ] | Pseudoalteromonas luteoviolacea | 100% | 0 | 76% |
|  | no_tag_f_2731 | WP_044617469 [enoyl-CoA hydratase] | Gynuella sunshinyii | 100% | 3,00E-115 | 61% |
|  | no_tag_f_2732 | WP_028981307  [enoyl-CoA hydratase] | Sporocytophaga myxococcoides | 98% | 7,00E-118 | 67% |
|  | no_tag_f_2734 | WP_007960100  [polyketide beta-ketoacyl:ACP synthase ] | Pelosinus fermentans | 97% | 1,00E-173 | 56% |
|  | no_tag_f_2735 | WP_045825692 [acyl-carrier-protein] | Teredinibacter sp. 991H.S.0a.06 | 100% | 1,00E-170 | 59% |
|  | no_tag_f_2736 | WP_009579380 [malonyl CoA-ACP transacylase] | Fulvivirga imtechensis | 97% | 2,00E-102 | 47% |
|  | no_tag_f_2739 | WP_015820018 [4'-phosphopantetheinyl transferase] | Teredinibacter turnerae | 88% | 6,00E-75 | 53% |
|  | no_tag_f_2746 | WP_019604951 [hypothetical protein] | Teredinibacter turnerae | 100% | 3,00E-159 | 84% |
