# Supplementary material for: The gill-associated microbiome is the main source of wood plant polysaccharide hydrolases and secondary metabolite gene clusters in the mangrove shipworm Neoteredo reynei
Source: PLoS One. 2018 Nov 14;13(11):e0200437. doi: 10.1371/journal.pone.0200437 (PMC6235255; doi:10.1371/journal.pone.0200437)
Supplement: S7 Table — * Coding sequence automated annotated by RAST server. † Resistance genes annotated according the CARD server and considering the default stringency cutoffs for perfect/strict and loosely annotations. ‡ Biosynthetic Gene Clusters (BGCs) annotated according to the antiSMASH server, under the bacterial version and using default settings. (DOCX) [file pone.0200437.s013.docx]

**Table S7: Cellvibrionaceae representative genomes information and resistome and BGCs annotations.**

| **Genome** | **Family** | **Patrick ID** | **Life Style** | **GC content (%)** | **Size (bp)** | **Total Genes*** | **Resistome**† | **BGCs‡** | |
| --- | --- | --- | --- | --- | --- | --- | --- | --- | --- |
|  |  |  |  |  |  |  |  | **Nrps_PKS_hybrids** | **Other** |
| **Saccharophagus degradans 2_40** | Cellvibrionaceae | 203122_15 | Free living | 45.8 | 5057531 | 4415 | 230 | 18 | 9 |
| **Congregibacter litoralis KT71** | Halieaceae | 314285_4 | Free living | 57.7 | 4344414 | 3919 | 196 | 0 | 5 |
| **Cellvibrio japonicus Ueda107** | Cellvibrionaceae | 498211_5 | Free living | 52 | 4576573 | 3909 | 227 | 18 | 11 |
| **Cellvibrio sp. OA_2007** | Cellvibrionaceae | 529823_3 | Free living | 47.7 | 4595379 | 4057 | 217 | 10 | 23 |
| **Gilvimarinus agarilyticus strain JEA5** | Cellvibrionaceae | 679259_3 | Free living | 53.2 | 4190841 | 3669 | 201 | 0 | 17 |
| **Gilvimarinus polysaccharolyticus strain YN3** | Cellvibrionaceae | 863921_5 | Free living | 49.4 | 3746057 | 3385 | 186 | 0 | 16 |
| **Simiduia agarivorans SA1 = DSM_21679** | Cellvibrionaceae | 1117647_4 | Free living | 55.9 | 4309711 | 3876 | 224 | 0 | 13 |
| **Simiduia agarivorans SA2 = DSM_21679** | Cellvibrionaceae | 1117647_6 | Free living | 55.9 | 4274427 | 3802 | 218 | 0 | 13 |
| **Dasania marina DSM_21967** | Spongiibacteriaceae | 1121374_3 | Free living | 47.4 | 4104668 | 3766 | 214 | 0 | 16 |
| **Gilvimarinus chinensis DSM_19667** | Cellvibrionaceae | 1121921_3 | Free living | 51.2 | 4067804 | 3719 | 188 | 0 | 20 |
| **Marinimicrobium agarilyticum DSM_16975** | Cellvibrionaceae | 1122194_3 | Free living | 57.9 | 4500118 | 3725 | 209 | 4 | 8 |
| **Marinimicrobium sp. LS-A18** | Cellvibrionaceae | 1381596_3 | Free living | 59.0 | 3815107 | 3180 | 200 | 0 | 9 |
| **Gammaproteobacteria bacterium MOLA455** | Porticoccaceae | 1411685_3 | Free living | 50.0 | 2605026 | 2383 | 112 | 0 | 3 |
| **Cellvibrio sp. pealriver** | Cellvibrionaceae | 1622269_3 | Free living | 47.6 | 4427922 | 3992 | 243 | 0 | 13 |
| **Candidatus Endobugula sertula isolate AB1_4** | Cellvibrionaceae | 62101_8 | Host associated | 41.6 | 3350348 | 3259 | 121 | 18 | 10 |
| **Teredinibacter turnerae T7901** | Cellvibrionaceae | 377629_3 | Host associated | 50.9 | 5193164 | 4488 | 233 | 65 | 18 |
| **Teredinibacter turnerae T8602** | Cellvibrionaceae | 1056817_3 | Host associated | 51.0 | 5097488 | 4449 | 235 | 52 | 17 |
| **Teredinibacter turnerae T8415** | Cellvibrionaceae | 1056818_3 | Host associated | 50.8 | 5156472 | 4583 | 233 | 46 | 20 |
| **Teredinibacter turnerae T8412** | Cellvibrionaceae | 1056819_3 | Host associated | 51.1 | 5142266 | 4469 | 239 | 50 | 20 |
| **Teredinibacter turnerae T7902** | Cellvibrionaceae | 1056820_3 | Host associated | 50.8 | 5383525 | 4690 | 266 | 59 | 17 |
| **Teredinibacter turnerae T8513** | Cellvibrionaceae | 1070977_3 | Host associated | 51.0 | 5264228 | 4591 | 243 | 58 | 21 |
| **Teredinibacter turnerae T8402** | Cellvibrionaceae | 1073969_3 | Host associated | 50.9 | 5160666 | 4493 | 237 | 52 | 21 |
| **Cellvibrio sp. BR** | Cellvibrionaceae | 1134474_3 | Host associated | 48.8 | 4850031 | 4280 | 258 | 0 | 17 |
| **Teredinibacter turnerae T0609** | Cellvibrionaceae | 1206095_3 | Host associated | 51.2 | 5066656 | 4531 | 240 | 39 | 16 |
| **Cellvibrio mixtus subsp. mixtus J3_8** | Cellvibrionaceae | 1209072_3 | Host associated | 46.7 | 5171890 | 4585 | 280 | 28 | 14 |
| **Teredinibacter sp. 1162T.S.0a.05** | Cellvibrionaceae | 1286364_3 | Host associated | 47.7 | 4404964 | 3804 | 216 | 10 | 7 |
| **Teredinibacter sp. 991H.S.0a.06** | Cellvibrionaceae | 1286365_3 | Host associated | 51.1 | 5279031 | 4594 | 238 | 66 | 21 |
| **Teredinibacter turnerae 1133Y.S.0a.04** | Cellvibrionaceae | 1313298_4 | Host associated | 51.2 | 5099533 | 4488 | 233 | 47 | 21 |
| **Marinagarivorans algicola Z1** | Porticoccaceae | 1513270_3 | Host associated | 45.1 | 4057827 | 3642 | 181 | 4 | 5 |
| **Agarilytica rhodophyticola_017_strain 017** | Cellvibrionaceae | 1737490_4 | Host associated | 41.0 | 6878829 | 5928 | 271 | 20 | 36 |
| **gills.bin.1** | Cellvibrionaceae | nested | Host associated | 51.6 | 4351088 | 3791 | 206 | 36 | 15 |
| **gills.bin.4** | Cellvibrionaceae | nested | Host associated | 53.2 | 5168684 | 4727 | 242 | 22 | 22 |

* Coding sequence automated annotated by RAST server.

† Resistance genes annotated according the CARD server and considering the defaut stringency cutoffs for perfect/strict and loosely annotations.

‡ Biosynthetic Gene Clustes (BGCs) annotated according to the antiSMASH server, under the bacterial version and using default setttings.
